# Targeted Variant Assessments of Human Endogenous Retroviral Regions in Whole Genome Sequencing Data Reveal Retroviral Variants Associated with Papillary Thyroid Cancer

**DOI:** 10.3390/microorganisms12122435

**Published:** 2024-11-27

**Authors:** Erik Stricker, Erin C. Peckham-Gregory, Stephen Y. Lai, Vlad C. Sandulache, Michael E. Scheurer

**Affiliations:** 1 Department of Molecular and Human Genomics, Baylor College of Medicine, Houston, TX 77030, USA; stricker@bcm.edu; 2 Department of Pediatrics, Baylor College of Medicine, Houston, TX 77030, USA; erin.peckham-gregory@bcm.edu; 3 Department of Head and Neck Surgery, Division of Surgery, The University of Texas MD Anderson Cancer Center, Houston, TX 77030, USA; 4 Bobby R. Alford Department of Otolaryngology Head and Neck Surgery, Baylor College of Medicine, Houston, TX 77030, USA; vlad.sandulache@bcm.edu; 5 Department of Molecular Virology and Microbiology, Baylor College of Medicine, Houston, TX 77030, USA; 6 Texas Children’s Cancer & Hematology Center, Houston, TX 77030, USA

**Keywords:** human endogenous retrovirus, HERV, *Alu* elements, retroelements, papillary thyroid cancer, anaplastic thyroid cancer, targeted variant analysis, whole genome sequencing, GWAS, in vitro

## Abstract

Papillary thyroid cancer (PTC) is one of the fastest-growing cancers worldwide, lacking established causal factors or validated early diagnostics. Human endogenous retroviruses (HERVs), comprising 8% of human genomes, have potential as PTC biomarkers due to their comparably high baseline expression in healthy thyroid tissues, indicating homeostatic roles. However, HERV regions are often overlooked in genome-wide association studies because of their highly repetitive nature, low sequence coverage, and decreased sequencing quality. Using targeted whole-genome sequence analysis in conjunction with high sequencing depth to overcome methodological limitations, we identified associations of specific HERV variants with PTC. Analyzing WGS data from 138 patients with PTC generated through The Cancer Genome Atlas project and 2015 control samples from the 1000 Genomes Project, we examined the mutational variation in HERVs within a 20 kb radius of known cancer predisposition genes (CPGs) differentially expressed in PTC. We discovered 15 common and 13 rare germline HERV variants near or within 20 CPGs that distinguish patients with PTC from healthy controls. We identified intragenic–intronic HERV variants within *RYR2*, *LRP1B*, *FN1*, *MET*, *TCRVB*, *UNC5D*, *TRPM3*, *CNTN5*, *CD70*, *RYR1*, *RUNX1*, *CRLF2*, and *PCDH1X*, and three variants downstream of *SERPINA1* and *RUNX1T1*. Sanger sequencing analyses of 20 thyroid and 5 non-thyroid cancer cell lines confirmed associations with PTC, particularly for MSTA HERV-L variant rs200077102 within the *FN1* gene and HERV-L MLT1A LTR variant rs78588384 within the *CNTN5* gene. Variant rs78588384, in particular, was shown in our analyses to be located within a POL2 binding site regulating an alternative transcript of *CNTN5*. In addition, we identified 16 variants that modified the poly(A) region in *Alu* elements, potentially altering the potential to retrotranspose. In conclusion, this study serves as a proof-of-concept for targeted variant analysis of HERV regions and establishes a basis for further exploration of HERVs in thyroid cancer development.

## 1. Introduction

**Figure 1:**
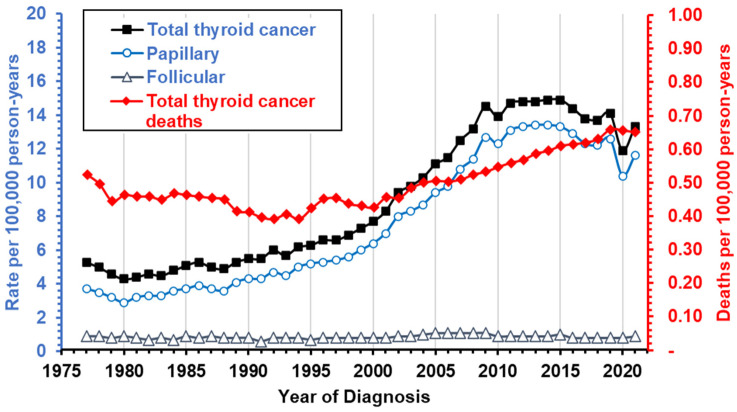
Trends in incidence of thyroid cancer overall and for histological subtypes. The number of new cases and deaths were obtained from the most recent SEER data (1975–2021) [10]. Incidence rates are marked by black or blue lines and correspond to the left y-axis, while mortality is marked in red and corresponds to the right y-axis.

Papillary thyroid cancer (PTC), accounting for 85% of all thyroid cancers [1], has been among the fastest-growing cancers worldwide, largely due to increased incidental detection, attributable to improved imaging and more sensitive diagnostic procedures [2]. In the US alone, incidence tripled between 1990 and 2020 [3], with rates plateauing between 13.7 and 14.9 per 100,000 person-years in the years since (Figure 1) [2,4]. While conservative diagnostics have slowed incidence, morbidity and mortality from late-stage and metastatic disease continue to rise, with death rates having increased by over 40% since 2000 [5,6,7,8,9]. Furthermore, thyroid cancers remain among the seven most common cancers in women, with 4 times higher prevalence in females under 50 compared to males [2]. In addition, thyroid cancer incidence varies by race/ethnicity, from 8.4 per 100,000 person-years in Non-Hispanic Black people to 15.5 in Non-Hispanic Asian populations [4].

Often asymptomatic, PTC is frequently diagnosed late, increasing metastasis risk (50%–75%) [1]. Despite high overall survival, treatment of PTC generates a substantial increase in morbidity (odds ratio = 2.56), and recurrent PTC, found in over 25% of patients, is frequently fatal, with 40–60% mortality in older (>45) adults [1,6,11]. PTC tumors can also unpredictably progress to anaplastic thyroid carcinoma, significantly decreasing survival [12]. To date, no established causes or early diagnostics exist for PTC, highlighting the need for novel risk factors and biomarker research. PTC, with its low somatic mutation density in exome sequences [13], serves as a suitable disorder for the initial study of the contributions of non-exonic sequences to cancer development. The identification of only seven genes with significantly different mutation frequencies in PTC [13] in the presence of over 400 cancer-related genes with significantly different expression levels suggests that variation in other elements, such as human endogenous retroviruses (HERVs), may contribute to disease [14].

Advances in whole-genome sequencing have enabled analysis of previously undetected HERVs, which comprise 8% of the genome, and their relationships to disease. Consequently, HERVs have been associated with numerous health outcomes, including several cancers [11,15,16,17,18,19,20,21,22,23,24,25,26,27]. While <1% of HERV loci have known functions [28,29], recent findings indicate their active involvement in beneficial functions, signifying a paradigm shift in thought about their contributions to health and disease. Some putative physiological functions include immune regulation [27,30,31], cell differentiation [32,33], cell fusion [34,35], and transcriptional regulation [36,37,38,39], all key hallmarks in cancer development. Studies of the beneficial roles of HERVs are of major importance, as many normal tissues—contrary to prevailing thought—express a significant number of HERV genes [40]. Uniquely, healthy thyroids express high baseline levels of HERVs similar to some tumors, indicating possible inactivation of beneficial HERV functions in PTC [40,41].

Furthermore, HERV long terminal repeats (LTRs), which flank the viral genes, harbor over 64% of all human-specific transcription factor binding sites (TFBSs) in human embryonic stem cells [42]. In many cancers, HERV LTRs have been observed to drive cancer-specific or tissue-specific transcripts, including oncogenes such as ADAM metallopeptidase with thrombospondin type 1 motif 5 (*ADAMTS5*), which is specifically controlled by the LTR mammalian LTR transposon 1J2 (MLT1J2) in thyroid tissues [43]. A study by Chang et al. (2019) linked somatic single nucleotide variants (SNVs) in HERV genes to various cancers, including thyroid cancer, while other analyses identified polymorphic HERV insertion sites uniquely associated with thyroid malignancies [44,45].

Even though HERVs have been observed to be polymorphic, HERV retrotransposition is controversial since sequences such as *Alu* elements in humans have higher abundance and mobility [46,47,48,49]. *Alu* elements belong to the class of short interspersed elements (SINEs). *Alu* elements are considered the most widespread transposable elements in the genome, with more than 1 million copies in the human genome [50]. Generally, *Alu* elements have a dimeric structure of around 300 bp length with two separate monomers that are connected by an A-rich linker region and are terminated by a 3′ poly(A)-tail [51]. For retrotransposition, the *Alu* sequence is amplified and reverse transcribed using an RNA polymerase III-derived transcript and the open reading frame 2 (ORF2) product from long interspersed element 1, which possesses endonuclease and reverse transcriptase activities [52,53,54,55]. Although the poly(A)-tail does not confer functions in the same sense as polyadenylated mRNAs, the A-rich region is believed to play important roles in the priming of reverse transcription and allows interactions with poly(A)-binding proteins [56].

Therefore, we set out to investigate the effects of variations in HERV and *Alu* elements on the development of PTC. To enrich the potential transcriptional effects of HERVs, we filtered whole-genome sequencing data of blood and tumor samples from 138 American patients with PTC for regions near or within differentially expressed cancer predisposition genes (CPGs) and performed targeted variant calling. We compared the resulting HERV genotype profiles with variant call data from 2015 controls [57,58] attributing to gender and ancestral population profiles. Variants that distinguished patients with PTC from healthy controls and suggested functional associations through in silico analyses were evaluated for representation in 20 thyroid and 7 non-thyroid cancer cell lines by Sanger sequencing.

## 2. Materials and Methods

### 2.1. The Cancer Genome Atlas and 1000 Genomes Project Data

We obtained paired-end DNA sequencing data from patients with PTC with corresponding metadata on patient gender, self-reported race and ethnicity, age, tumor stage, and vital status from The Cancer Genome Atlas (TCGA) database [59] using the Genomic Data Commons Data Portal, dbGaP study accession: phs000178.v11.p8 [60]. The whole-genome sequencing (WGS) data available included thyroid tumors and matched normal blood samples for a total of 138 patients with PTC. We acquired the WGS files in binary alignment map (BAM) file format and then converted them into a compressed reference-oriented alignment map (CRAM) file format for storage using cramtools (version 3.0) [61], selecting options for ignoring tags OQ:CQ:BQ, capturing all other tags, implementing 8-binning of the Illumina quality scores, and preserving read names. We indexed the CRAM files with SAMtools (version 1.6) [62]. Tumor tissues and blood samples from 12 individuals were sequenced with a high average coverage of 40× in addition to a low average coverage of 4× sequencing run. In our analyses, we excluded the low-coverage WGS data with both available low- and high-coverage data. Whole-genome sequencing files without high-coverage reruns and only low-coverage data were not excluded. For all TCGA data analyses, we utilized the b37 human reference FASTA file (Homo_sapiens_assembly19.fa, MD5sum: 886ba1559393f75872c1cf459eb57f2d, accessed on 25 April 2019). The average coverage of the available WGS files was assessed with SAMtools coverage and depth commands [62].

We retrieved the 1000 Genomes Project (1KGP) variant call files (VCFs) directly from the University of California Santa Cruz data resource (https://hgdownload.cse.ucsc.edu/gbdb/hg19/1000Genomes/, accessed on 26 November 2019) [57,58]. To allow the merging of TCGA and 1KGP variant call data, we applied Genome Analysis Tool Kit’s (GATK) UpdateVCFSequenceDictionary with the b37 human reference FASTA file to the 1KGP variant call files [63] and indexed the resulting variant files with BCFtools (version 1.9) [62].

Both the PTC patient cohort and the 1KGP control cohort were divided into a training (60%) and validation (40%) set through multi-trial randomization comparisons, ensuring equivalent ratios between different sequencing coverages (where available), genders, age (where available), ancestral population (self-reported), and superpopulation.

### 2.2. Identification of HERVs Near or Within Differentially Expressed Cancer Predisposition Genes

Cancer predisposition genes (CPGs) were defined as any gene conferring moderately or highly increased risk of cancer in children or adults. We assembled a list of unique CPGs from four different sources: a review by Rahman (2014) [63], a primary research article by Zhang et al. (2015) [64], the Network of Cancer Genes [65], and the Catalogue Of Somatic Mutations In Cancer (COSMIC) Cancer Gene Consensus [66]. We used TCGA-derived mRNA sequencing data available through the BioXpress database (version 2.0) for differential expression in cancer to identify CPGs of interest that were significantly differentially up- or downregulated (|log2 fold change| > 1, adjusted *p*-value < 0.05) in patients with PTC [67,68].

### 2.3. Targeted Variant Discovery in TCGA Data

We used the GATK version 4.1.2.0 according to recommended workflows to identify germline SNVs and indels in the WGS data obtained from TCGA (see Supplemental Figure S1) [69]. In short, we applied GATK’s pipeline in Genomic VCF (GVCF) and BP_RESOLUTION mode to the HERV regions near or within differentially expressed CPGs for each CRAM file in the training sets, resulting in a GVCF file for each sample. We applied the quality scores from the recalibration with a truth sensitivity of 99.0%. We added corresponding rsIDs to the detected variants, utilizing both the dbSNP genotypes from the resource bundle [70] and the Kaviar genomic variant database [71]. In the final step, variants with a variant quality score (VQS) below 90.0 were separated. TCGA tumor and blood, as well as high- and low-coverage samples, were analyzed separately.

When we tried to apply the GATK pipeline to WGS data from the 1KGP, we encountered problems with reference genome compatibilities between the TCGA and 1KGP samples, not allowing us to joint call the samples. Therefore, we decided to rely on the separate joint calling of TCGA and 1KGP sample cohorts by comparing our variant call data from the TCGA samples with the variant call file provided by the 1KGP. It should be mentioned at this point that by choosing this approach, we were no longer able to differentiate the absence of a HERV locus from a genotype that matches the reference in the 1KGP dataset. Therefore, we initially chose variants with MAF > 0 in the 1KGP dataset, circumventing this issue of indeterminable HERV locus presence. In particular, we filtered the 1KGP VCF file for variants in HERV regions near or within differentially expressed CPGs using the same pipeline described above for the TCGA WGS data and then combined them with the TCGA variant call data using BCFtools (version 1.9) merge function with the missing_to_ref option, which sets any unseen genotype to ref (0/0) after normalizing both VCF files to the same hg19 human reference genome, splitting multiallelic sites into biallelic records [62]. 

### 2.4. Determination of Ancestral Populations

We determined genetic ancestry for all cases and controls using Structure software (version 2.3.4) [72,73] on the basis of germline (DNA from blood samples) genotypes at 85/179 ancestral informative markers (AIMs) [74] available through HapMap samples (CEU, YRI, CHB/JPT, MEX), which served as our reference ancestral populations. European (EUR), African (AFR), and East Asian (EAS) individuals were defined as having >90% EUR genetic ancestry, ≥70% AFR ancestry, and ≥70% EAS ancestry, respectively. Hispanic Americans (HIS) were defined as individuals for whom the percentage of AmerIndian (Native American) genetic ancestry was ≥10% and greater than the percentage of African or East Asian ancestry [75]. Individuals whose ancestral distribution did not fit these thresholds were classified as Admixed American (AMR). Since the PTC samples were obtained from an American patient cohort without evident representation of South Asian (SAS) ancestry, we excluded individuals from the 1KGP control cohort assigned to the SAS superpopulation. This was further supported by the observation that 485/489 (99%) of all SAS individuals were classified as HIS when using AIMs.

### 2.5. Statistical Analysis

We conducted statistical genotype comparisons using a logistic regression model computed with Plink (version 1.90 beta-5.3) [76,77]. Unadjusted statistical models were generated without the addition of covariates, minor allele count (MAC), or minor allele frequency (MAF) filters. Multivariable logistic regression analyses were performed with the addition of gender and ancestry as covariates. For the incorporation of ancestry profiles, we used the continuous principal component assignments to EUR, AFR, EAS, and HIS ancestry generated by the Structure software [72,73]. To assess the effect size, we calculated the odds ratios (ORs) from the beta coefficients of the additive multivariate models. Then, we calculated the OR differences for each variant by acquiring the absolute difference between the multivariate model OR and the unadjusted model OR and then dividing it by the OR from the unadjusted model. Results from the multivariate regression analyses were visualized using a Manhattan graph plotting the negative logarithmic *p*-value against the chromosome location of each variant. We evaluated the linkage between variants using vcftools’ geno-r2 function (version 0.1.16) [78]. Statistical analyses of Sanger sequencing results were conducted in R using a fitted logistic regression model generated with the glm() function and family = ‘binomial’ parameter [79].

### 2.6. Functional in Silico Predictions

We evaluated and visualized specific gene expression across various healthy tissues using the Genotype-Tissue Expression Portal (GTExPortal) facilitated by the Broad Institute Consortium [14]. The GTExPortal includes samples from 54 non-diseased tissue sites across nearly 1000 individuals, primarily collected for molecular assays, including WGS, whole exome sequencing (WES), and RNA-Seq [14]. The data used for the analyses described in this study were obtained from GTEx Analysis Release V8 (dbGaP Accession phs000424.v8.p2) on 17 May 2023. Transcription factor and protein binding predictions were evaluated using the HaploReg (version 4.1) [80,81] and RegulomeDB databases [82]. Protein binding was based on Encyclopedia of DNA Elements (ENCODE) data, while histone marks were on Epigenome Roadmap data as described in Ward and Kellis (2016) [81]. Predicted chromatin states were derived from ChromHMM analyses based on ENCODE ChIP-seq data, including eight histone modifications [82,83]. Chromatin accessibility, splice site detection, and genomic context of variants were further assessed with the University of California Santa Cruz (UCSC) genome browser [58]. Gene ontology analyses were conducted using the GOnet interactive gene ontology tool (http://tools.dice-database.org/GOnet/, accessed on 30 May 2023) [84]. Based on the functional predictions, variants from each of the following three categories were selected: (1) associated CPG with high expression in healthy thyroid tissue and significantly lower expression in PTC; (2) variation affects predicted polymerase or enhancer protein binding; and (3) location within the *Alu* element. *p*-values for differential expression of CPGs were obtained using the OncoMX portal [85]. Isoform expression profiles for TCGA RNA datasets were obtained from the GEPIA2 web server (http://gepia2.cancer-pku.cn/, accessed on 31 May 2023) [86]. The RNA-Seq datasets for GEPIA2 are based on the University of California, Santa Cruz (UCSC) Xena project (http://xena.ucsc.edu, accessed on 31 May 2023) [87].

### 2.7. Chemicals and Reagents

Cell culture reagents were purchased from either Gibco, Thermo Fisher Scientific, Inc. (Waltham, MA, USA), Sigma-Aldrich (St. Louis, MO, USA), or Corning (Corning, NY, USA) (see Supplemental Table S1). Enzymes, reagents, chemicals, and kits for DNA processing, as well as all primers in the form of standard tube oligos, were purchased from Thermo Fisher Scientific, Inc. (Waltham, MA, USA) or New England Biolabs, Inc. (Ipswich, MA, USA).

### 2.8. Cell Culture

Thyroid cancer cell lines were provided by Dr. Stephen Lai at The University of Texas MD Anderson Cancer Center, Houston, TX; head and neck cancer cell lines by Dr. Vlad Sandulache, Baylor College of Medicine (BCM), Houston, TX; melanoma cell lines by Dr. Albert Ribes-Zamora at the University of St. Thomas, Houston, TX; and liver cancer cell lines by Dr. Betty Slagle at BCM, Houston, TX (see Supplemental Table S2). We cultured the cells in either RPMI 1640, supplemented with 10% FBS, 2 mM L-glutamine, 1 mM sodium pyruvate, 50 μg/mL streptomycin, and 50 U/mL penicillin, supplemented with 10% FBS, 1× Nonessential Amino Acids (NEAA), 2 mM L-glutamine, 50 μg/mL streptomycin, and 50 U/mL penicillin, or DMEM, supplemented with 10% FBS, 4.5 g/L D-Glucose, L-Glutamine, 50 μg/mL streptomycin, and 50 U/mL penicillin at 37 °C in a humidified incubator with 5% CO_2_. We subcultured the cells every 3–5 days using Dulbecco’s Phosphate Buffered Saline (DPBS) for an initial wash and 0.25% Trypsin 2.21 mM EDTA to detach the cells from the culture dish. We confirmed cell authenticity through short tandem repeat (STR) profiling by the Cytogenetic and Cell Authentication Core of the MD Anderson Cancer Center, Houston, TX (for results, see Supplemental Table S3).

### 2.9. Genomic DNA Extraction

Cells were grown in a 10 cm dish to a confluence of 80–90% at 37 °C in a humidified incubator with 5% CO_2_ and lysed by the addition of QIAamp lysis buffer (catalog # 19075, QIAGEN, LLC, Germantown, MD, USA) after two washes with DPBS solution. Subsequently, cell lysates were harvested with a cell scraper and transferred into a tube prepared with proteinase K. Genomic DNA (gDNA) was extracted using the QIAamp DNA mini kit (catalog # 51304, QIAGEN, LLC, Germantown, MD, USA) according to the manufacturer’s protocol.

### 2.10. Targeted Genotyping by Sanger Sequencing

We obtained genomic sequences in the context (±2000 bp) of each evaluated variant or variant pair from the UCSC genome browser with its “Get DNA in Window” function (https://genome.ucsc.edu/cgi-bin/hgc?o=37130799&g=getDna, accessed on 30 August 2022) [58]. We designed specific primers using Primer3 (v4.1.0) (https://primer3.ut.ee/, accessed on 30 August 2022) [88] and checked for the presence of potential SNVs using Genetools SNPCheck V3 (https://genetools.org/SNPCheck/docs.htm, accessed on 30 August 2022) (see Supplemental Table S4). We excluded off-target binding using PrimerBlast (accessed on 30 August 2022) [59,89]. We amplified regions of 422–2032 bp surrounding variants rs10179937 and rs200077102 within the Fibronectin 1 (*FN1*) gene, rs10925366 and rs10802602 within the Ryanodine Receptor 2 (*RYR2*) gene, rs10166768 within the LDL Receptor Related Protein 1B (*LRP1B*) gene, rs78588384 within the Contactin 5 (*CNTN5*) gene, rs13246949 upstream of the Rap Associating With DIL Domain (*RADIL*) gene/downstream of Monocyte To Macrophage Differentiation Associated 2 (*MMD2*), rs1987574 and rs78393784 downstream of the Serpin Family A Member 1 (*SERPINA1*) gene using conventional end-point polymerase chain reaction (PCR). We performed the PCR in 3–4 times 20 μL reaction volume using 50 ng of gDNA, 2 μL of Phusion Plus Buffer, 0.4 μL 10 mM dNTPs, 1 μL forward and reverse primers each, and 0.7 μL of Phusion Plus polymerase according to the manufacturer’s instructions. Annealing temperatures and extension time of the PCR protocols were adjusted for each amplicon (see Supplemental Table S5), while 30 s of 98 °C initial denaturation, 10 s of 98 °C denaturation, 10 min of 98 °C final extension, and 34 cycles were used for all PCR reactions. We confirmed amplicon sizes with either 1% SYBR Safe E-gels, 1.2% ethidium bromide (EtBr) Precast Agarose E-gels, 2% EtBr Precast Agarose E-gels, or manually cast 1.5% EtBr gels. We purified the resulting PCR products with the GeneJet PCR purification kit. We analyzed the purified PCR fragments through Sanger di-deoxy nucleotide sequencing by the GeneWiz sequencing center (GeneWiz, Azenta Life Sciences, Burlington, MA, USA). To determine the genotype for each cell line, we evaluated the Sanger sequencing chromatograms for the presence of single (homozygosity) or double (heterozygosity) peaks using the APE plasmid editor (version 3.1.1) [90].

### 2.11. Visualization and Data Processing

We visualized the tabularized data using the ggplot2 (version 3.3.6) R package [80] in conjunction with the Cairo R package (version 1.5–15) [81]. We used the dplyr (version 1.0.9) [82], stringr (version 1.4.0) [83], tidyverse (version 2.0.0) [84], and vcfR (version 1.14.0) [85] packages to aid in data processing and analysis. We generated Manhatten plots with CMplot (version 4.3.1) [86]. All scripts were executed on R version 4.2.0 [79].

## 3. Results

### 3.1. Cancer Predisposition Genes (CPGs) in PTC Include a Total of 3725 HERV Sequences Within or in Close Proximity

To identify genomic regions of oncogenic significance, we extracted 2884 different CPGs from Rahman (2014) [63], a primary research article by Zhang et al. (2015) [64], the Network of Cancer Genes [65], and the COSMIC Cancer Gene Consensus [66] (Supplemental Figure S2, Supplemental Table S6). Through evaluation of TCGA mRNA data for thyroid cancer patients from BioXpress, we identified 117 CPGs that were differentially expressed in PTC (log2FC > 1 or log2FC < −1). Our list of CPGs included proto-oncogenes (e.g., *FOS*, *JUN*, and *SOX11*), tumor suppressor genes (e.g., *ADAMTS9*), and DNA repair genes (e.g., *E2F1*). Since HERVs are known to be enriched in transcriptional regulatory elements [87] and therefore carry higher potential to be the cause for CPG dysregulation, we extracted 3725 unique HERVs within a 20-Kbp radius of 107 CPGs (10 CPGs had no reported HERV sequences near or within) using the EnHERV database [88]. For further analyses, the HERV loci were summarized into 2866 non-overlapping genomic regions (see Supplemental Table S7). Genes Runt-Related Transcription Factor 1 (*RUNX1*), *LRP1B*, *CNTN5*, EPH Receptor A6 (*EPHA6*), Sidekick Cell Adhesion Molecule 1 (*SDK1*), Protocadherin 11 X-Linked (*PCDH11X*), *RYR2*, ALK Receptor Tyrosine Kinase (*ALK*), Receptor Potential Cation Channel Subfamily M Member 3 (*TRPM3*), and P21 (RAC1) Activated Kinase 7 (*PAK7*) were the ten CPGs most enriched for HERV sequences. The total size of the HERV sequences was 3,321,253 bp with a median size of 882 bp (25% quartile = 707 bp, 75% quartile = 1022 bp) (see Supplemental Figure S3). While 2812 HERVs were located intronic to CPGs, only 8 sequences were located in an exon, namely HERV-L MLT1E2 in *C6orf118*, LTR37 in EPH Receptor A3 (*EPHA3*), HERV-L MLT1J in Beta-1,4-Mannosyl-Glycoprotein 4-Beta-N-Acetylglucosaminyltransferase (*MGAT3*), HERV-L MSTA in *PCDH11X*, HERV-L MLT1H2 in *RADIL*, LTR41B in Ras Association Domain Family Member 6 (*RASSF6*), and two HERV-L MLT2A1 in *ACSM2A*. Of the extragenic HERV sequences, 494 were located upstream of CPGs and 411 downstream. Additionally, 72 complete HERVs and 3380/3725 (97%) soloLTRs were located within 20 kbp of CPGs.

### 3.2. Targeted Variant Calling Revealed 612,603 High-Quality Variants Within CPG-Associated HERV Regions

We obtained paired-end WGS data for a total of 125 blood samples and 138 matched tumor samples from patients diagnosed with PTC from The Genome Cancer Atlas (TCGA) (Table 1). To generate two distinct datasets and avoid overfitting, we divided the files into a training set containing sequencing data from 83 (60%) individuals and a validation set comprising 55 (40%) with samples from 64 (77.1%) females and 19 (22.9%) males for data training and 42 (76.4%) females and 13 (23.6%) males for validation. Using the 3725 HERV sequences located within 20 Kbp of CPG regions, we performed initially targeted variant calling with the Genome Analysis Tool Kit (GATK) [69] on the training blood and tumor samples, resulting in information for 3,693,035 genome locations. We attained a total of 612,603 high-quality variants after Variant Quality Score Recalibration, which uses machine learning to model the technical profile of true variants in the HapMap 3.3, OMNI 2.5, and 1000 G phase 2.5 training resource to filter out probable artifacts from the callset. To conserve variants for later fine-mapping efforts [89] as well as HERV regions with repeats and low coverage [90], we decided against the application of read depth or minor allele frequency (MAF) filters for variant calling.

### 3.3. Multivariate Analyses Revealed Strong Confounding Effects of Gender and Ancestral Profile on HERV Variants

To account for ancestral population- and gender-driven heterogenicity particularly present in HERV loci [91], we compared TCGA genotype data for tumor and blood samples separately with variant call data from the 1KGP [57] using a multivariate logistic regression model with population ancestry and sex as covariates (independent variables). Although we did not expect gender to affect autosomal HERV variation, we included gender as a covariate to account for differences in the number of X chromosomes. Overall, the 1KGP dataset contained SNVs and indels from 2015 healthy adults, who were divided into a testing set of 1224 (60%) individuals and a validation set of 791 (40%), similar to the TCGA data preserving equal gender and ancestral superpopulation ratios (see Table 2). As a result, the training set included 300 individuals assigned to the European (EUR) superpopulation, 216 individuals to the Admixed American (AMR) superpopulation, 408 to the African (AFR) superpopulation, and 300 to the East Asian (EAS) superpopulation. While ancestry information was present for all 1KGP controls, self-reported information on race and ethnicity in the TCGA dataset was 25% incomplete or inconclusive. Therefore, we employed 85 ancestry-informing markers (AIMs) and performed principal component analyses with HapMap samples (CEU, YRI, CHB/JPT, MEX) as reference [74]. We assigned either European (EUR), African (AFR), East Asian (EAS), Hispanic American (HIS), or Admixed American (AMR) ancestry to each sample based on their genetic ancestry predominance and ratios (see Supplemental Table S8). This resolved all but four of the 36 individuals in the PTC cohort with “no reported” race/ethnicity by assigning them to an ancestral population other than AMR. However, 22/83 (26%) patients with PTC had less than 80%, and 15/83 (18%) patients had less than 70% assignment to an ancestral population, indicating significant admixture that could interfere with the genomic evaluation of HERVs. Therefore, we used continuous assignments to EUR, HIS, AFR, EAS, and AMR in our multivariate analyses (Supplemental Table S8, Supplemental Table S9). To access comparable covariates for healthy controls, we assigned ancestral profiles to the 1KGP cohort using the same 85 AIMs and principal components.

We observed strong confounding effects (evaluated as a >10% change in the odds ratio (OR) between unadjusted and multivariate models) for gender and ancestral population profiles for 97.9% of the variants. When comparing results from our unadjusted and multivariate logistic regression models of the training set, we saw equally strong effects on statistically significant variants (see Supplemental Figure S4). Of all blood variants with significant *p*-values in either the unadjusted, multivariate logistic regression model or both, 210/240 (87.5%) were strongly affected by adjustment for gender and ancestry, while 30/240 (12.5%) variants displayed minor differences in ORs, indicating no gender- or genetic ancestry-driven variance. Statistical comparison of PTC tumor sample variants with healthy controls yielded similar results of 252/267 (94.3%) variants displaying confounding and 15/267 (5.6%) exhibiting only small changes in ORs.

### 3.4. Evaluation of Common Variants Exposed 15 HERV Variants Significantly Different in Frequency Between PTC and Healthy Controls

**Figure 2:**
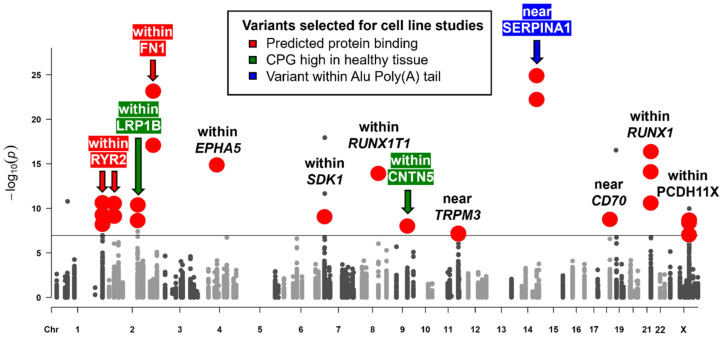
Germline variants within HERV elements near or within CPGs. The x-axis represents chromosomes, while the y-axis shows the transformed (−log10(p)) *p*-value obtained from our multivariate logistic regression models. The significance threshold is 7.09 (−log10(8.07 × 10^−8^)) according to the number of variants analyzed. Solid large red circles indicate variants significant in PTC blood and tumor samples in the training and validation set, while smaller grey circles above the threshold line could not be confirmed with the same statistical significance in at least one of the sample sets. Arrows designate variants chosen for in vitro analyses based on functional in silico analyses. Red arrows and highlighting indicate variants affecting predicted protein binding sites. Green arrows and highlighting signify associated CPGs with high expression levels in healthy tissues and reduced transcription in PTC. The blue arrow and highlighting denote variants within the poly(A)-tail of an *Alu* element.

The training set comparisons of the 1KGP variant call data with variant call data obtained from TCGA PTC blood and tumor samples using the GATK pipeline in GVCF mode resulted in 38 significantly different HERV variants, intronic to 13 and close to 4 distinct CPGs (see Figure 2). We confirmed 26/38 HERV variants in the validation set (Supplemental Table S10). All 26 variants have been identified as common variants, defined as exceeding an MAF of 5% in the general population. A total of 21/26 variants were located in CPG introns, while variants near *MMD2/RADIL*, *RUNX1T1*, *SERPINA1*, and *CD70* were located between 507 bp and 17,531 bp from the nearest CPG. When comparing our results from the unadjusted logistic regression model and our multivariate logistic regression model, 20/26 variants were unaffected by gender and ancestral profile in the training and validation set, while rs2618671 and rs2779420 within *RYR2*, rs200093832 within Transient *TRPM3*, rs370565365 within Acyl-CoA Synthetase Medium-Chain Family Member 2A (*ACSM2A*), rs112385920 downstream of *CD70*, and rs13046555 within *RUNX1* displayed significance only after adjustment. When assessing minor allele frequencies in PTC samples comparing training and validation sets, we noticed inconsistent distributions for rs7682763 within EPH Receptor A5 (*EPHA5*), variants rs370565365 within *ACSM2A*, and rs13046555 within *RUNX1*, excluding them from further analyses. Furthermore, we omitted variant rs10956571 within Adenylate Cyclase 8 (*ADCY8*) from further evaluations because the variant displayed significance only in the PTC tumor training set and PTC blood validation set. Although variants rs10166768 (C>G) with *LRP1B* and rs200093832 within *TRPM3* reached a *p*-value below the significance threshold only in the PTC tumor samples and not the PTC blood samples compared to the 1KGP controls, MAFs did not suggest somatic mutations.

While we did not have access to variant calls and metadata of other healthy control sets, we compared overall minor allele frequencies (MAFs) from our studies with the data from the Genome Aggregation Database (GnomAD) (Table 3) [92]. Interestingly, we observed large variations in reported frequencies between the 1KGP and the GnomAD for eight variants (including the already excluded variant rs370565365 within ACSM2A), leaving a total of 15 variants for further assessments. Variant rs10166768 within *LRP1B* was not rejected on the basis of 1KGP and GnomAD discrepancies since its tri-allelic nature left results inconclusive.

### 3.5. Rare Variants Affect the Poly(A)-Tail Length of Several Alu Elements

**Figure 3:**
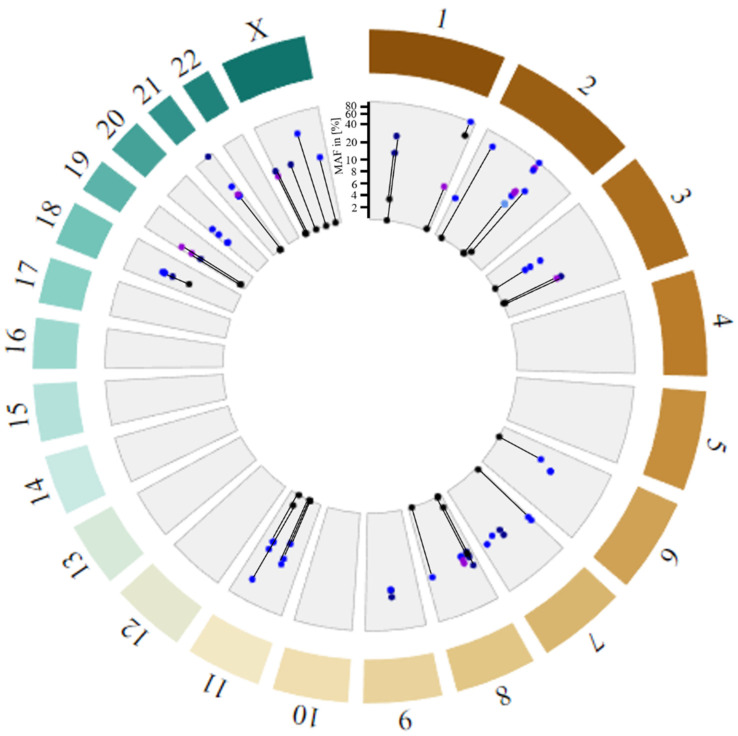
Distribution of variants within retroelements with minor allele frequency (MAF) > 10% in PTC samples and 0% or not reported in 1KGP data. GnomAD MAFs (black); MAFs of PTC samples for variants in HERVs (blue), *Alu* element poly(A)-tails (dark blue), *Alu* element linkers (light blue), and all others (violet). Variants with matching PTC samples and GnomAD MAFs are connected by black lines. Note that allele frequencies are plotted on a semi-logarithmic scale (linear: 0–10%; logarithmic: 10–100%).

In addition to the evaluation of common variants, we assessed HERV variants absent in the 1KGP VCFs, i.e., with an assigned MAF of 0%. A limitation of a logistic regression model is that dependent variable probabilities have to fall between 0 and 1. Hence, no output can be generated for variants with an overall MAF of exactly 0. Overall, genotype calls were available for all variants from at least 80/138 PTC tumor samples and 74/125 PTC blood samples, with an average of ten individuals with undetermined genotype (./.). Since a sample size of 74 allowed the detection of a 10% difference with a statistical power of 81%, we decided to extract all variants with MAF ≥ 10% in the PTC samples and compared them to 1KGP 30× coverage and GnomAD control cohorts (Figure 3, Supplemental Table S11). We detected a total of 71 rare variants (defined as MAF ≤ 1% in controls), of which 28 variants had at least a 10 times higher MAF frequency in PTC blood samples compared to the GnomAD-reported MAF. In our list of rare variants, 38 loci had variant frequencies reported neither for the 1KGP nor the GnomAD samples. We detected eight variants with slightly higher MAF in PTC tumor samples relative to PTC blood samples, suggesting potential somatic mutations. Interestingly, putative somatic variants rs1166234155 (MAF_blood_ = 0.05; MAF_tumor_ = 0.09) and rs1272563337 (MAF_blood_ = 0.03; MAF_tumor_ = 0.09) 3124 bp downstream of ADAM Metallopeptidase With Thrombospondin Type 1 Motif 9 (*ADAMTS9*) were in linkage disequilibrium (R^2^ = 0.85), as were variants rs373561192, rs1303831387, rs1353282464, and rs1406672069 (MAF_blood_ = 0.02–0.09; MAF_tumor_ = 0.12) within the HERV9 LTR12C located in exon 3 of P21 (RAC1) Activated Kinase 5 (*PAK5*) (R^2^ = 0.89–1).

While most variants were located in HERV sequences within CPG introns, 21/71 variants could be found in *Alu* elements (see Supplemental Table S12). Variants within Leucine Rich Repeat Containing 7 (*LRRC7*), Tumor Suppressor Candidate 3 (*TUSC3*), and *TRPM3* were detected to be in the poly(A)-tail of the respective *Alu* elements with their G>A or C>A mutations extending the poly(A)-tail from 29 to 37 by the *LRRC7* variants, from 20 to 26 by the *TUSC3* variants, and 25 to 36 by the *TRPM3* variants.

For our additional in silico functional analyses, we concentrated on the common alleles designated significant in the logistic regression analyses. In this way, we ensured adjustment of ancestry and gender in the statistical assessment of the frequency differences and excluded erroneous non-detection rather than low incidence numbers for variants as a cause for false positives. Read depth for all common variants was, on average, 40× for high- and 4× for low-coverage WGS data, which matched the overall read depth and therefore confirmed technical accuracy. Although HERVs in intronic regions have been reported to carry the capacity to alter gene splicing and express viral genes or long-noncoding RNAs (lncRNAs), we focused our functional evaluations on transcriptional effects. For this reason, we also enriched regulatory units beforehand by selecting regions near differentially expressed CPGs.

Protein binding and transcription factor binding motifs potentially affected by the variants were assessed using the HaploReg (v4.1) [93,94] and RegulomeDB databases (see Table 4) [95]. For all variants 2–9, transcription factor binding motifs were present in their corresponding regions with the exception of variants within the *PCDH11X* gene on chromosome X. Sequence regions affected by variants rs1987574 and rs78393784 downstream of *SERPINA1* and rs112385920 downstream of *CD70* displayed a particular enrichment with 7–9 predicted TFBSs, while variants rs10802602 within the *RYR2* gene, rs10179937, and rs200077102 within the *FN1* gene were located within YY1 and POL2 binding sites, respectively, supported by ChIP-Seq experiments. Furthermore, ENCODE chromatin state data showed strong transcription in parathyroid adenomas and quiescent chromatin in the non-malignant thyroid gland at the *FN1* variant sites. Overall, enhancer histone marks (H3K4me1, H3K27ac) and promoter histone modifications (H3K4me3, H3K9ac) were detected in various blood, brain, breast, epithelial, heart, lung, mesenchymal, muscle, and stem cell lines for the majority of the variant regions, yet thyroid cell line data were not available.

**Figure 4:**
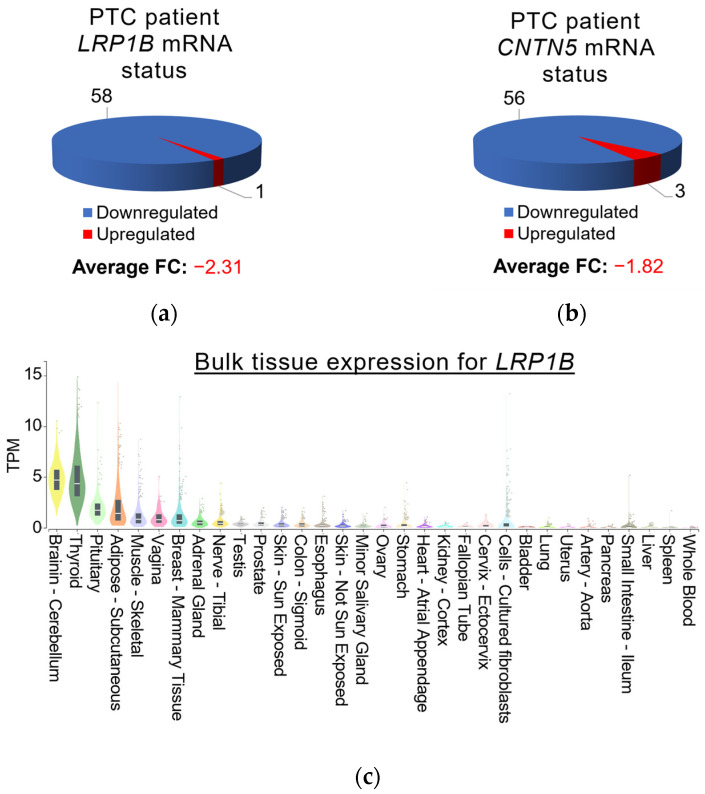

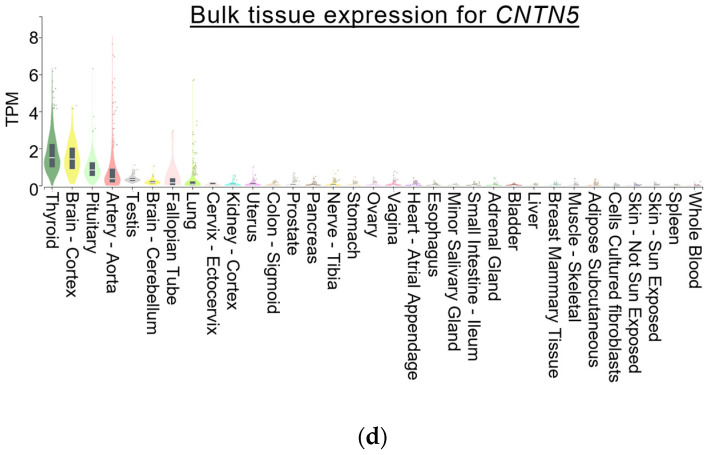
*LRP1B* and *CNTN5* mRNA expression in patients with PTC and normal tissues. Expression data, including fold change (FC) for (**a**) *LRP1B* and (**b**) *CNTN5* mRNA in PTC patients from the TCGA, were obtained through the BioXpress database browser [67,68], while mRNA levels in healthy tissues for (**c**) *LRP1B* and (**d**) *CNTN5* presented in TPM (transcripts per million) were derived from the Genotype-Tissue Expression (GTEx) portal [14]. The sum of all TPM values is similar in all samples, so that a TPM value signifies a relative expression level, in principle allowing the comparison between samples [96].

To assess the degree of transcriptional changes in the variant-associated CPGs, we compared TCGA PTC RNA-Seq data accessed through the BioXpress database browser [67,68] with RNA levels in healthy tissues obtained from the Genotype-Tissue Expression (GTEx) portal [14]. Overall, *FN1*, *SERPINA1*, *CD70*, and *RUNX1* mRNA levels were upregulated in 81.4–93.2% of the 59 evaluated patients with PTC, whereas *RYR2*, *LRP1B*, *RUNX1T1*, *TRPM3*, *CNTN5*, and *PCDH11X* mRNA expression was found to be significantly downregulated (see Supplemental Table S13). While most CPGs are expressed to similar degrees in several healthy tissues (see Supplemental Figure S5), normal *LRP1B* and *CNTN5* expression in thyroid tissues stood out, as only healthy brain tissues displayed similarly high mRNA levels (see Figure 4). Combined with the significant reduction in *LRP1B* and *CNTN5* transcripts in PTC samples, central roles in thyroid homeostasis conferred by these two genes are suggested. Comparing overall CPG transcript levels in healthy thyroid tissues, *FN1* (TPM = 86) mRNA levels were shown to be the highest, followed by *SERPINA1* (TPM = 21) and *LRP1B* (TPM = 16).

**Table 4:** Results of in silico functional predictions obtained using the HaploReg v4.2 and RegulomeDB databases.

Name	CPG	REF>ALT	Protein Bound ^a^	Motifs ^b^	Chromatin State	Histone Mark ^c^
rs10802602	RYR2	C>G	YY1 CEBPA, CEBPB, CEBPG	PAX-8, THAP1, YY1	-	Enhancer
rs2618671	RYR2	C>G	-	AHR, KLF9	Hetero-chromatin	Promoter, Enhancer
rs2779420	RYR2	C>T	-	EGR1, FOXP1, RREB1	-	-
rs10166768	LRP1B	C>T,G	-	SOX4, SOX15	-	Enhancer
rs10179937	FN1	T>A	POL2	FOXP1, KLF9, RREB1	Strong transcription	Promoter, Enhancer
rs200077102	FN1	T>A	POL2	FOXP1, RREB1, SOX3, SOX15	Strong transcription	Promoter, Enhancer
rs12543616	RUNX1T1	G>A	-	EWSR1, IRF1, STAT1, STAT2	-	Enhancer
rs200093832	TRPM3	A>G	-	EP300, EWSR1-FLI1, IRF1, HDAC2, PRDM1, SPI1	-	Enhancer
rs78588384	CNTN5	G>C	-	ATF7, FOXP1, IRF1, RREB1, SPI1	-	Promoter
rs1987574	SERPINA1	T>A	-	CUX1, EP300, EVI1, FOXP1, HDAC2, HMGA1, HOMEZ, IRF1–4, ZNF35, ZNF384	monocyte eQTL	Enhancer
rs78393784	SERPINA1	T>A	-	EP300, EVI1, FOXP1, HDAC2, HOMEZ, IRF1, POU6F1, ZNF35, ZNF384	-	Enhancer
rs112385920	CD70	C>T	-	EWSR1-FLI1, HDAC2, SP1, SPZ1, STATTCF12, ZNF143, ZNF263	Weak Repressed polyComb	Promoter, Enhancer
rs2076859	RUNX1	T>C	-	SMAD2, SMAD3	-	Promoter
rs2754876	PCDH11X	G>C	-	BCL6B	-	-
rs2750652	PCDH11X	A>G	-	-	-	Enhancer

^a^: Proteins bound in ChIP-Seq experiments [97], ^b^: For affected protein binding motifs, a set of positional weight matrix was collected from TRANSFAC, JASPAR, protein-binding microarray (PBM), and ENCODE ChIP-seq experiments [98,99,100,101], ^c^: chromatin state segmentations (15-state and 25-state) from the Roadmap Epigenomics Project.

**Figure 5:**
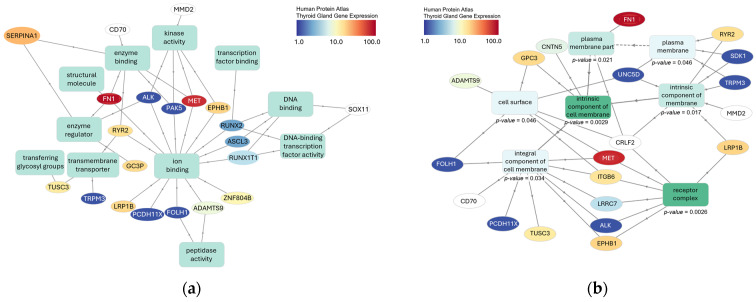

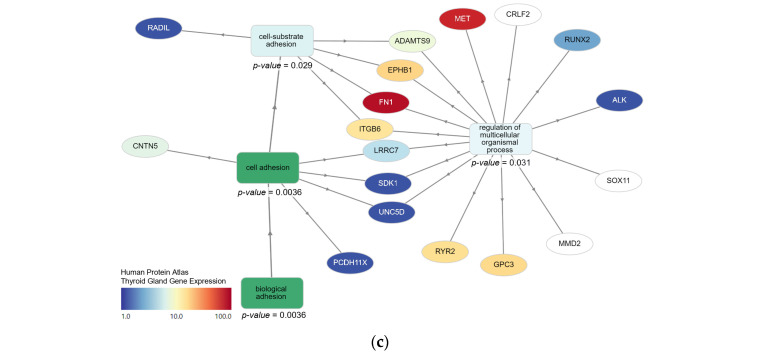
Graphical representation of gene ontology analyses performed with the GOnet tool. GO term enrichment analyses were performed for (**a**) biological processes, (**b**) cellular components, and (**c**) molecular functions separately based on the Gene Ontology (GO, http://geneontology.org/, accessed on 30 May 2023) database [102]. Solid lines represent an “is_a” relationship, while dashed lines indicate a “part_of” relationship read in the direction of the arrow. All indicated *p*-values were computed using a Fisher exact test and FDR adjusted, i.e., represent q-values.

We conducted gene ontology studies of the CPGs potentially affected by common and rare variants using the GOnet interactive gene ontology tool. In our evaluation of molecular functions, we discovered an enrichment in ion-binding proteins associated with 13/36 submitted genes (see Figure 5a). Other moderately enriched functions included kinase activity (5/36 genes) and DNA binding (4/36 genes). Our assessment of cellular locations revealed a significant enrichment of intrinsic components of the plasma membrane (11/36 genes, *p*-value (False discovery rate (FDR) adjusted) = 0.0029) and receptor complexes (7/36, *p*-value (FDR adjusted) = 0.0029) (see Figure 5b), which also matched our observed enrichment in cell adhesion molecules (10/36, *p*-value (FDR adjusted) = 0.036) (see Figure 5c). Genes potentially associated with cell migration and cancer metastasis included *CNTN5*, *FN1*, Integrin Subunit Beta 6 (*ITGB6*), *EPHB1*, Unc-5 Netrin Receptor D (*UNC5D*), *SDK1*, *RADIL*, *LRRC7*, *PCDH11X*, and *ADAMTS9*. Furthermore, gene set enrichment analysis revealed associations of *LRP1B* and *RYR2* with thyrotoxic periodic paralysis.

Cancer stage comparison yielded no clear association with specific variants (Supplemental Figure S6). At the time of evaluation, 98% of the PTC patients were alive. Accordingly, no survival analyses were possible.

### 3.6. In Vitro Analyses of Thyroid Cancer Cell Lines Mirrored Low Variant Frequencies for rs200077102 Within FN1 and rs78588384 Within CNTN5 Detected in PTC Samples

**Figure 6:**
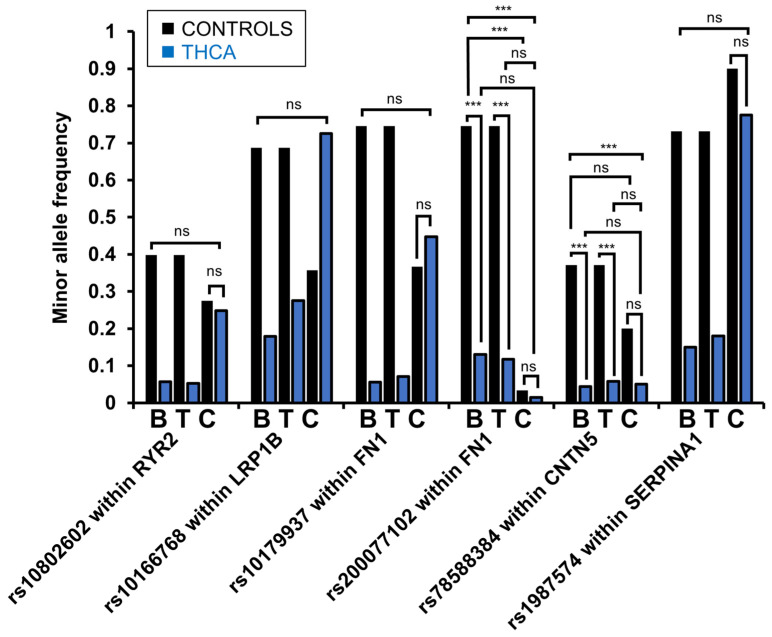
Minor allele frequencies of selected variants in whole-genome sequencing data and cell lines. MAFs were compared between PTC blood (B; n = 125), PTC tumor (T; n = 138), and 1KGP control samples (n = 2015), thyroid (n = 20), and non-thyroid cancer (n = 5; n_rs10166768_ = 7) cell lines (C) in a logistic regression analysis (***: *p* ≤ 0.001; ns: not significant). All WGS differences between PTC and 1KGP samples were statistically significant (*p* ≤ 0.001), while the statistics not shown were not significant.

To confirm the in vitro relevance and establish model systems for the study of the variants detected, we evaluated the presence of six variants in thyroid and non-thyroid cancer cell lines. Therefore, we obtained genomic DNA from seven PTC cell lines (MDA-T22, MDA-T32, MDA-T41, MDA-T68, MDA-T85, MDA-T120, and TPC-1), three poorly differentiated thyroid carcinoma (PDTC) cell lines (MDA-T171, MDA-T189, and MDA-T192), ten ATC cell lines (MDA-T178, MDA-T187, MDA-T220, MDA-T245, MDA-T248, MDA-T269, MDA-T273, U-HTH7, U-HTH83, and U-HTH104), and seven non-thyroid cancer cell lines (CaSki, SiHa, C33A, HN30, UM-SCC47, A375, HepG2). We selected variants to be evaluated each based on one of the following in silico functional predictions: (1) rs10166768 within *LRP1B* and rs78588384 within *CNTN5* because of the high expression of the associated CPG in healthy thyroid tissue compared to other tissues and significantly lower expression in PTC; (2) variants rs10802602 within *RYR2*, rs10179937, and rs200077102 within *FN1* because they affect predicted polymerase or enhancer protein binding; and (3) rs1987574 downstream of *SERPINA1* because of its location within an *Alu* element. While MAFs of each variant observed in 1KGP samples were confirmed to be similar in the GnomAD database, rs10166768 within *LRP1B* was included in the analyses despite 1KGP and GnomAD discrepancies since its tri-allelic nature resulted in inconclusive observations. We amplified regions of 422–2032 bp surrounding the selected variants and assessed the genotypes for each cell line based on chromatograms obtained through Sanger sequencing. We calculated minor allele frequencies for each variant and assessed statistical differences using a logistic regression model (see Figure 6). For both variants, rs78588384 within *CNTN5* and rs200077102 within *FN1*, low MAFs, similar to the MAFs detected in PTC blood and tumor samples, were observed (*p* ≤ 0.001). No statistical differences were found between control cancer cell lines and thyroid cancer cell lines for rs78588384 and rs200077102. Variant rs10166768 within *LRP1B* displayed an MAF in thyroid cancer cells similar to the 1KGP samples and in the control cancer cell lines equivalent to the GnomAD reported genotypes, suggesting substantial population heterogeneity.

### 3.7. The Genomic Context of rs200077102 and rs78588384 Indicates Transcriptional Dysregulation Caused by the Variants

**Figure 7:**
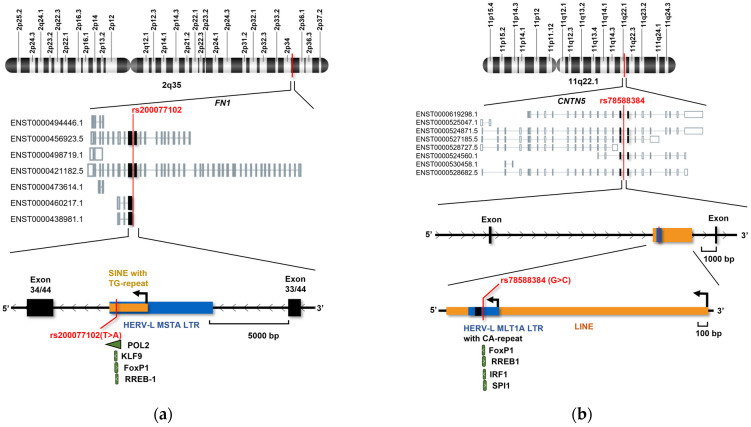
Genomic context of variants confirmed by Sanger sequencing of thyroid cancer cell lines. The first level displays the location of (**a**) variant rs200077102 within chromosome 2 and (**b**) variant rs78588384 within chromosome 11. The second level shows different isoforms obtained from the GTEx portal [14]. The last levels illustrate the functional context of the variants. Predicted TFBSs affected by the variant are marked in green.

Variant rs200077102 is located in a MIR3 short interspersed nuclear element (SINE) retrotransposon, which is inserted into the HERV-L MSTA LTR within the long arm of chromosome 2 (Figure 7). Both the SINE and *FN1* genes are on the negative strand. The elements are situated 411 bp upstream of exon 34 from the longest *FN1* isoform. All *FN1* isoforms are expressed in the brain, spleen, and heart but not expressed in healthy thyroid tissue. Variant rs200077102 is 411 bp away from the *FN1* exon 34 (exon 1 for some isoforms). Interestingly, variant rs200077102 is positioned in the promoter region of the non-protein-coding ENST00000460217.1 and protein-coding 241 amino acid long ENST00000438981.1 *FN1* isoforms. Isoform expression profiles for TCGA cancers revealed significantly higher expression of ENST00000460217.1 in PTC (TPM = 4.08) compared to normal thyroid (TPM = 0.25), while ENST00000438981.1 expression was only reported in HCCs (TPM_tumor_ = 0.39; TPM_normal_ = 1.4) and not evaluated in PTCs (see Supplemental Figure S7). Furthermore, high levels of ENST00000460217.1 could be detected in sarcomas (TPM_tumor_ = 3.1; TPM_normal_ = 1), healthy bile ducts (TPM = 3.2), and healthy lung tissues (TPM_normal_ = 3.43; TPM_LUAD_ = 1.03; TPM_LUSC_ = 0.72).

The CNTN5 variant rs78588384 was located in a HERV-L MLT1A LTR inserted into the long interspersed nuclear element 2a (LINE-2a) within the long arm of chromosome 11. Both the LINE and HERV LTR are on the negative strand, while the *CNTN5* is transcribed from the plus strand. The variant rs78588384 is 3808 bp away from the nearest *CNTN5* exon and just upstream of a CA-repeat region. *CTNT5* generates multiple isoforms with four major transcripts. Isoform ENST00000619298.1 is predominantly expressed in healthy brain and thyroid tissues, while ENST00000525047.1, ENST00000524871.5, and ENST00000528727.5 are primarily expressed in the healthy thyroid. Interestingly, ENST00000619298.1 was significantly reduced in PTC patients (TPM_normal_ = 0.29; TPM_tumor_ = 0.05), in addition to patients with glioblastoma (TPM_normal_ = 1.15; TPM_tumor_ = 0.23) and low-grade glioma (TPM_normal_ = 1.15; TPM_tumor_ = 0.13) (see Supplemental Figure S8). Furthermore, isoforms ENST00000525047.1 (TPM_normal_ = 0.43; TPM_tumor_ = 0), ENST00000524871.5 (TPM_normal_ = 0.29; TPM_tumor_ = 0), and ENST00000528727.5 (TPM_normal_ = 0.06; TPM_tumor_ = 0) decreased in levels.

## 4. Discussion

In our targeted whole genome sequencing analyses approaches, we focused on regions with putative functions, i.e., HERV sequences, that are enriched in transcriptional elements and viral gene products, therefore decreasing noise-to-signal ratios [87]. As a result, statistical comparison between PTC cases and controls revealed 15 common germline HERV variants significantly different in frequency between the two cohorts. For our discovery and selection of PTC-specific variants, we performed functional in silico analyses. Based on predicted protein binding sites, transcriptional levels of the respective CPGs, and cellular context, we selected six variants for evaluation in thyroid cancer cell lines. Using 20 thyroid cancer and 7 control cancer cell lines, we discovered that variants rs200077102 within *FN1* and rs78588384 within *CNTN5* displayed comparable low MAF in vitro as observed in the PTC blood and tumor samples. Both *FN1* and *CNTN5* are cell adhesion molecules at the plasma membrane, as shown in our gene ontology studies, which indicated general enrichment of variants in plasma membrane-bound cell adhesion molecules with receptor and ion-binding functions [103,104].

FN1 has been demonstrated to affect matrix remodeling indirectly through membrane-bound signaling molecules such as Transforming Growth Factor Beta (TGFβ) via SMADs, RET, and ERK [105] or directly through Phosphoinositide 3-Kinase (PI3K)/AKT [102]. Furthermore, FN1 has been shown to control cell survival, proliferation, and epithelial–mesenchymal transition (EMT) in cancers [102]. In thyroid cancer cells, silencing of *FN1* significantly reduced proliferation, adhesion, migration, and invasion [106]. HERV LTRs have the ability to function as cryptic promoters, promoting the expression of alternative isoforms. For instance, in tissues from patients with diffuse large B-cell lymphoma (DLBCL), LTR2 activity drives the formation of a chimeric isoform of the Fatty Acid-Binding Protein 7 (*FABP7*) gene [107]. Accordingly, our data suggest that the alternative isoform ENST00000438981.1 of *FN1* is potentially expressed by the upstream MIR3 SINE or HERV-L MSTA LTR. Although there was no thyroid cancer data available, ENST00000438981.1 expression was reported in hepatocellular carcinomas. Additionally, POL2 binding to the region affected by the rs200077102 variant was shown by chromatin immunoprecipitation in the human hepatocellular carcinoma cell line HepG2 [108], providing a link between the transcript and the presented functional region. Promoter onco-exaptation, as suggested for the variant MIR3 SINE or HERV-L MSTA LTR, has been observed for many cancers, e.g., *IRF5* driven by the demethylated LOR1a LTR in HL [109,110]. It should be noted that other *FN1* transcripts (Supplementary Figure S7) were also upregulated in PTC patients, which potentially indicates enhancer functions of the variable region.

In contrast to *FN1*, downregulation of *CNTN5* was shown to be associated with tumor metastasis [111]. Interestingly, while significantly decreased in PTC, *CNTN5* has been observed even further downregulated in the more aggressive follicular variant of PTC [111]. Generally, Contactins are GPI-anchored proteins involved in neuronal development, while CNTN5 contributes to axonal targeting, synaptic formation, and plasticity [104]. Even though associations between *CNTN5* mutations and neurological disorders have been shown, molecular functions of the molecule are still poorly understood [104]. Our studies revealed that variant rs78588384 within *CNTN5* could enhance the expression of a competing transcript, a lncRNA, or miRNA, since HERV LTRs have the ability to act as cryptic promoters and confer tissue-specific activation [112], e.g., *ADAMTS5*, which is specifically controlled by the LTR MLT1J2 in thyroid tissues [43]. Alternatively, rs78588384 could prevent the binding of an enhancer or induce a transcriptional repressor protein. Therefore, transcriptional activity changes conferred by mutational variants require confirmation through reporter luciferase assays, and transcription factor binding could be evaluated with an electrophoretic mobility shift assay. HERV LTRs have also been shown to contain splice donors, which leads to the induction of alternative splicing [109]. The presence of splice variants could be best assessed by RT-qPCR in future analyses [113].

The assessment of variants without reported MAF in 1KGP samples revealed a total of 28 rare variants with at least 10 times higher MAF in PTC samples compared to the GnomAD-reported MAF. Notably, several of the rare variants (MAF < 5%) were in linkage disequilibrium and affected *Alu* element poly(A)-tail length. Roy-Engel et al. (2002) demonstrated that the average length of *Alu* element poly(A)-tails in the human genome is between 21 and 26 bp, while the poly(A)-tails of very recent disease-causing *Alu* insertions were observed to be between 40 and 97 bp in length [56]. In our analyses of rare variants, we identified several variants in linkage disequilibrium, which extended the *Alu* element poly(A)-tails by 6–11 bps. We postulate that this could potentially lead to the reactivation of *Alu* elements with retrotransposition capabilities. Such retrotransposition, especially when integrated within oncogenes, could further drive tumor oncogenesis in general and thyroid cancer development specifically. Furthermore, repeat elements in the genome introduced by retrotransposition have been shown to contribute to changes in the three-dimensional structure of the DNA and genomic instability, a hallmark of cancer [114]. However, single nucleotide polymorphisms, as observed in the ERVs, are less likely to induce instability unless they are associated with the binding sites of architectural proteins, such as *CTCF* [115].

While our study provides valuable insights into genomic associations of HERVs and PTC, several limitations should be considered when interpreting the results. Due to the limited metadata available for our whole genome sequencing samples, we cannot exclude the potential influence of environmental, demographic, or behavioral factors on the outcomes. Future studies could benefit from large databases, such as the recently published by the All of Us Research Program [116], which provides whole genome sequencing data linked with medical and survey data, enabling the investigation of additional cofactors. For our functional analyses, we lacked thyroid-specific ENCODE chromatin and protein binding data [94], in addition to thyroid-specific expression quantitative trait loci (eQTLs) data. Therefore, functional predictions from non-thyroid cells were used as proxies. However, we were able to incorporate a substantial number of thyroid cancer cell lines for in vitro studies, which will also be available for future functional assays.

Our analyses underscored the importance of adjusting for covariates since confounding for gender and ancestry was evident. Our approach of continuous ancestral assignments, first described by Halder et al. (2008) [74], allowed us to measure admixture within individuals contributed by all of their ancestors rather than one parental line [56], the inference of admixture dynamics [117,118,119], and the separation of genetic and environmental effects and, therefore, the study of underlying biologic effects [56]. We recognize that this methodology also has its limitations. Ancestral influences on variation can be local to a region of the genome, and our approach assigns (even though more granular) ancestral profiles for the whole individual and not specific chromosome sections. This can lead to faulty adjustments, particularly for individuals with strong admixture or ancestry-dependent local variation in disease-related regions. While currently restricted by computational limitations, future investigations of genomic variants should consider local ancestry inference (LAI) methods such as *Tractor* [120] to disentangle disease-associated variants from ancestral variability.

For our SNV/indel detection, the GATK pipelines demonstrated high accuracy (F-scores > 0.99) across numerous benchmark datasets [121,122]. Additionally, it provided short processing times enabled by initial separate variant calling, joint variant calling results, and simple integration of adjustments for reruns. Our targeted variant calling enabled rapid multiple comparisons and the identification of common and rare variants [122]. We detected 71 rare variants; for example, variant rs986066503 within *RUNX1* was undeterminable (genotype: ./.) in 48/125 (38.4%) blood and 54/138 (39.1%) tumor samples, and variant rs78999285 within the *Alu* element in *TUSC3* was undefined in 60/125 (48.0%) blood and 62/138 (44.9%) tumor samples. These variants would have been generally excluded from GWAS analyses, emphasizing the higher sensitivity of our targeted approach.

## 5. Conclusions

In this study, we describe the first attempt to identify HERV-related genetic risk markers for PTC. We identified several SNVs within HERVs within or near cancer predisposition genes (CPGs) with elevated PTC risk scores. In addition, we were able to validate two variants in thyroid cancer cell lines and predict transcriptional regulatory consequences to their presence. Overall, this study provides a proof-of-concept for targeted variant assessment of HERV regions and lays a foundation for further investigations of HERVs in thyroid oncogenesis.

## Figures and Tables

**Table 1:** Demographic characteristics and sample properties of individuals diagnosed with PTC.

	Training Set (*n* = 83; 60%) ^1^	Validation Set (*n* = 55; 40%) ^1^
**Blood samples**		
Low coverage only	50 (64.1%)	32 (68.1%)
High coverage only	20 (25.6%)	11 (23.4%)
High and low coverage	8 (10.3%)	4 (8.5%)
**Tumor samples**		
Low coverage only	52 (62.7%)	36 (65.5%)
High coverage only	23 (27.7%)	15 (27.3%)
High and low coverage	8 (9.6%)	4 (7.3%)
**Gender**		
Female	64 (77.1%)	42 (76.4%)
Male	19 (22.9%)	13 (23.6%)
**Age at diagnosis**		
Average age	48.68	48.06
**Race/Ethnicity** **(self-reported)**		
Non-Hispanic White	51 (61.4%)	32 (58.2%)
Hispanic White	2 (2.4%)	3 (5.5%)
Black or African American	3 (3.6%)	2 (3.6%)
Asian	5 (6%)	4 (7.3%)
Not reported	22 (26.5%)	14 (25.5%)
**Ancestral population** **(calculated)**		
EUR	44 (53%)	31 (56.4%)
HIS	24 (28.9%)	14 (25.5%)
AFR	2 (2.4%)	1 (1.8%)
EAS	3 (3.6%)	3 (5.5%)
AMR	10 (12%)	6 (10.9%)
**Vital status**		
alive	81 (97.6%)	54 (98.2%)
dead	2 (2.4%)	1 (1.8%)
**Tumor stage**		
I	43 (51.8%)	31 (56.4%)
II	9 (10.8%)	11 (20%)
III	19 (22.9%)	7 (12.7%)
IV	11 (13.3%)	6 (10.9%)
Not reported	1 (1.2%)	0 (0%)

^1^ TGCA samples were distributed in a 3:2 ratio into a training and validation set. AFR, African; AMR, Ad Mixed American; EAS, East Asian; EUR, European; HIS, Hispanic.

**Table 2:** Demographic characteristics and ancestral profiles of individuals in the control cohort.

	Training Set (*n* = 1224; 60%) ^1^	Validation Set (*n* = 791; 40%) ^1^
**Gender**		
Female	765 (50.8%)	506 (50.9%)
Male	740 (49.2%)	489 (49.1%)
**Superpopulation**		
EUR	300 (19.9%)	203 (20.4%)
AMR	216 (14.4%)	131 (13.2%)
AFR	408 (27.1%)	253 (25.4%)
EAS	300 (19.9%)	204 (20.5%)
**Ancestral population** **(calculated)**		
EUR	206 (13.7%)	147 (14.8%)
AMR	126 (8.4%)	75 (7.5%)
AFR	397 (26.4%)	242 (24.3%)
EAS	301 (20%)	204 (20.5%)
HIS	194 (12.9%)	123 (12.4%)

^1^ TGCA samples were distributed in a 3:2 ratio into a training and validation set. AFR, African; AMR, Ad Mixed American; EAS, East Asian; EUR, European; HIS, Hispanic.

**Table 3:** Minor allele frequencies of common variants. The table outlines the HERV variants detected significantly different between PTC tumor or PTC blood samples and 1KGP healthy controls in training and validation sets with the addition of data from the Genome Aggregation Database (GnomAD).

Name	CPG	MAF PTC Blood	MAF PTC Tumor	MAF 1KGP	MAF GnomAD
rs10802602	*RYR2*	3.8%	3.3%	39.6%	22.3%
rs2618671	*RYR2*	32.5%	30.9%	57.1%	56.7%
rs2779420	*RYR2*	30.1%	29.3%	53.6%	48.3%
rs13030271 ^◊^	*LRP1B*	8.2%	11.5%	37.8%	0.0%
rs10166768 (C>T)	*LRP1B*	17.9%	23.0%	67.7%	17.9%
rs10166768 (C>G)	*LRP1B*	50.0%	32.1%	0%	49.3%
rs10179937	*FN1*	8.8%	7.8%	74.9%	NA
rs200077102	*FN1*	7.6%	6.5%	74.9%	41.5%
rs7682763 ^†^	*EPHA5*	25.8%	25.0%	71.6%	NA
rs13311049 ^◊^	*SDK1*	9.9%	10.9%	38.9%	16.4%
rs13311637 ^◊^	*SDK1*	6.7%	6.3%	50.8%	2.4%
rs611655 ^◊^	*MMD2 (RADIL)*	0.5%	2.5%	43.8%	0.0%
rs12543616	*RUNX1T1*	18.9%	22.4%	76.0%	48.7%
rs10956571 ^‡^	*ADCY8*	25.3%	24.0%	62.9%	55.3%
rs200093832	*TRPM3*	26.7%	18.9%	53.5%	71.8%
rs61909780 ^◊^	*CNTN5*	2.7%	2.5%	38.8%	14.1%
rs78588384	*CNTN5*	4.5%	5.5%	36.9%	29.8%
rs1987574	*SERPINA1*	20.0%	26.8%	73.1%	NA
rs78393784	*SERPINA1*	38.6%	31.2%	29.4%	NA
rs370565365 ^†,◊^	*ACSM2A*	5.3%	NA	25.2%	2.0%
rs112385920	*CD70*	50.7%	51.3%	79.2%	81.6%
rs2076859	*RUNX1*	8.1%	11.7%	82.7%	NA
rs3989120 ^◊^	*RUNX1*	22.0%	21.4%	82.7%	0.0%
rs13046555 ^†^	*RUNX1*	15.0%	22.2%	32.1%	46.2%
rs778825437	*PCDH11X*	3.8%	3.3%	39.6%	NA
rs2754876	*PCDH11X*	18.7%	20.7%	65.0%	36.9%
rs2750652 ^◊^	*PCDH11X*	39.2%	40.0%	73.0%	35.7%

Minor allele frequencies (MAFs) are color-coded, with red indicating common, blue, and rare variants and saturation lowest and highest values. †: variants excluded from further analyses based on inconsistent distribution in PTC tumor, PTC blood, training, and validation datasets; ^‡^: variant excluded from further analyses since significant results from the PTC tumor training set could not be confirmed in PTC tumor validation set, and results from PTC blood validation set could not be confirmed in PTC blood testing set; ◊: variants excluded from further analyses based on 1KGP and GnomAD MAF discrepancies; NA: variant was not detected in our cases or GnomAD.

## Data Availability

The data that support the findings of this study are available as Supplementary Tables or on request from the corresponding author, M.E.S. The data are not publicly available due to their containing information that could compromise the privacy of research participants.

## Supplementary Materials

The following supporting information can be downloaded at: https://www.mdpi.com/article/10.3390/microorganisms12122435/s1, Table S1. Chemicals and Reagents; Table S2. Cell line source and culture media; Table S3. MD Anderson STR profiling results; Table S4. Primer table; Table S5. PCR annealing and extension times and temperatures table; Table S6. Cancer predisposition genes with papillary thyroid cancer expression data. Table S7. HERVs near cancer predisposition genes differentially expression in papillary thyroid cancer. Table S8. TGCA THCA patient metadata, including demographic and clinical data. Table S9. 1KGP gender and ancestry data, training and validation subset assignment; Table S10. Chromosomal locations of variants with significantly different frequencies in PTC tumor or PTC blood samples compared to 1KGP healthy controls; Table S11. Rare variants with significantly different frequencies in the TGCA data compared to gnomAD and non-detection in 1KGP; Table S12. CPG expression in PTC from BioXpress and OncoMX. Table S13. Minor allele frequencies of rare variants within Alu elements are significantly different between PTC tumor or PTC blood samples and the Genome Aggregation Database (GnomAD); Figure S1. Variant calling pipeline scheme; Figure S2. Extraction of HERVs within 20 Kbp radius of differentially expressed CPGs in PTC; Figure S3. Size distribution of HERVs near or within CPGs; Figure S4. Distribution of odds ratio (OR) changes for variants with significant *p*-values from the training set; Figure S5. RYR2, FN1, RUNX1T1, SERPINA1, CD70, RUNX1, and PCDH11X mRNA expression in patients with PTC and normal tissues; Figure S6. Minor allele frequencies of different variants in TGCA PTC tumor samples according to stage; Figure S7. Expression profiles of different FN1 isoforms; Figure S8. Expression profiles of different *CNTN5* isoforms.
