# Supplementary material for: Targeted Variant Assessments of Human Endogenous Retroviral Regions in Whole Genome Sequencing Data Reveal Retroviral Variants Associated with Papillary Thyroid Cancer
Source: Microorganisms. 2024 Nov 27;12(12):2435. doi: 10.3390/microorganisms12122435 (PMC11679660; doi:10.3390/microorganisms12122435)
Supplement: Supplementary file 1 [file microorganisms-12-02435-s001.zip › microorganisms-3263285_supplements.pdf]

Supplemental materials

Supplemental Table S1. Chemicals and Reagents.

Chemical/Reagent	Catalog #	Supplier
1% SYBR Safe E-gels	16314628	Thermo Fisher Scientific, Inc. (Waltham, MA, USA)
1.2% ethidium bromide (EtBr) Precast Agarose E-gels	G501801	Thermo Fisher Scientific, Inc. (Waltham, MA, USA)
100 bp DNA ladder	15628019	Thermo Fisher Scientific, Inc. (Waltham, MA, USA)
2% EtBr Precast Agarose E-gels	G501802	Thermo Fisher Scientific, Inc. (Waltham, MA, USA)
Agarose	17850	Thermo Fisher Scientific, Inc. (Waltham, MA, USA)
DMSO	67-68-5	Sigma-Aldrich (St. Louis, MO, USA)
dNTPs	N0447S	New England Biolabs Inc (Ipswich, MA, USA)
DPBS	D8537	Sigma-Aldrich (St. Louis, MO, USA)
EDTA	25-025-CI	Corning (Corning, NY, USA)
Ethanol	64-17-5	Sigma-Aldrich (St. Louis, MO, USA)
Ethidium bromide	15585011	Thermo Fisher Scientific, Inc. (Waltham, MA, USA)
Fetal bovine serum (FBS)	A31605	Gibco, Thermo Fisher Scientific, Inc. (Waltham, MA, USA)
GelPilot DNA Loading Dye, 5x	239901	QIAGEN, LLC (Germantown, MD, USA)
GeneJet PCR purification kit	K0702	Thermo Fisher Scientific, Inc. (Waltham, MA, USA)
High glucose Dulbecco's Modified Eagle Medium (DMEM)	11965092	Gibco, Thermo Fisher Scientific, Inc. (Waltham, MA, USA)
Isopropanol	67-63-0	Sigma-Aldrich (St. Louis, MO, USA)
L-glutamine	59202C	SAFC Biosciences, Sigma-Aldrich (St. Louis, MO, USA)
Nonessential Amino Acids (NEAA)	25-025-CI	Corning (Corning, NY, USA)
Phusion Plus polymerase	F630L	Thermo Fisher Scientific, Inc. (Waltham, MA, USA)
RPMI 1640	R8758	Sigma-Aldrich (St. Louis, MO, USA)
Sodium pyruvate	11360070	Gibco, Thermo Fisher Scientific, Inc. (Waltham, MA, USA)
Streptomycin/penicillin	15140122	Gibco, Thermo Fisher Scientific, Inc. (Waltham, MA, USA)
TAE buffer	24710030	Invitrogen, Thermo Fisher Scientific, Inc. (Waltham, MA, USA)
UltraPure Distilled Water	10977-015	Invitrogen, Thermo Fisher Scientific, Inc. (Waltham, MA, USA)

Supplemental Table S2. Cell line source and culture media.

	Primary site[a]	Age	Sex	Culture media	Source	Primary source
MDA-T22	PTC	79	F	RPMI 1640, 10% FBS, 2mM L-Glu, 1 mM Na-Pyruvate, 50u/ml Pen/Strep	Dr. Stephen Lin, MD Anderson, Houston, TX	Dr. Stephen Lin, MD Anderson, Houston, TX
MDA-T32	PTC	74	M	RPMI 1640, 10% FBS, 2mM L-Glu, 1 mM Na-Pyruvate, 50u/ml Pen/Strep	Dr. Stephen Lin, MD Anderson, Houston, TX	Dr. Stephen Lin, MD Anderson, Houston, TX
MDA-T41	PTC	74	M	RPMI 1640, 10% FBS, 2mM L-Glu, 1 mM Na-Pyruvate, 50u/ml Pen/Strep	Dr. Stephen Lin, MD Anderson, Houston, TX	Dr. Stephen Lin, MD Anderson, Houston, TX
MDA-T68	FVPTC	75	M	RPMI 1640, 10% FBS, 2mM L-Glu, 1 mM Na-Pyruvate, 50u/ml Pen/Strep	Dr. Stephen Lin, MD Anderson, Houston, TX	Dr. Stephen Lin, MD Anderson, Houston, TX
MDA-T85	PTC	61	M	RPMI 1640, 10% FBS, 2mM L-Glu, 1 mM Na-Pyruvate, 50u/ml Pen/Strep	Dr. Stephen Lin, MD Anderson, Houston, TX	Dr. Stephen Lin, MD Anderson, Houston, TX
MDA-T120	PTC	72	F	RPMI 1640, 10% FBS, 2mM L-Glu, 1 mM Na-Pyruvate, 50u/ml Pen/Strep	Dr. Stephen Lin, MD Anderson, Houston, TX	Dr. Stephen Lin, MD Anderson, Houston, TX
TPC-1	PTC	Adult	F	RPMI 1640, 10% FBS, 2mM L-Glu, 1 mM Na-Pyruvate, 50u/ml Pen/Strep	Dr. Stephen Lin, MD Anderson, Houston, TX	Dr. Junji Tanaka, Kanazawa Univ., Kanazawa, Japan
MDA-T171	PDTC	-	F	RPMI 1640, 10% FBS, 2mM L-Glu, 1 mM Na-Pyruvate, 50u/ml Pen/Strep	Dr. Stephen Lin, MD Anderson, Houston, TX	Dr. Stephen Lin, MD Anderson, Houston, TX
MDA-T189	PDTC	-	F	RPMI 1640, 10% FBS, 2mM L-Glu, 1 mM Na-Pyruvate, 50u/ml Pen/Strep	Dr. Stephen Lin, MD Anderson, Houston, TX	Dr. Stephen Lin, MD Anderson, Houston, TX
MDA-T192	PDTC	65	F	RPMI 1640, 10% FBS, 2mM L-Glu, 1 mM Na-Pyruvate, 50u/ml Pen/Strep	Dr. Stephen Lin, MD Anderson, Houston, TX	Dr. Stephen Lin, MD Anderson, Houston, TX

(a): papillary thyroid cancer (PTC), follicular variant of papillary thyroid carcinoma (FVPTC), poorly differentiated thyroid carcinoma (PDTC).

Supplemental Table S2. Cell line source and culture media (continued).

	Primary site[a]	Age	Sex	Culture media	Source	Primary source
MDA-T178	ATC	78	F	RPMI 1640, 10% FBS, 2mM L-Glu, 1 mM Na-Pyruvate, 50u/ml Pen/Strep	Dr. Stephen Lin, MD Anderson, Houston, TX	Dr. Stephen Lin, MD Anderson, Houston, TX
MDA-T187	ATC	74	F	RPMI 1640, 10% FBS, 2mM L-Glu, 1 mM Na-Pyruvate, 50u/ml Pen/Strep	Dr. Stephen Lin, MD Anderson, Houston, TX	Dr. Stephen Lin, MD Anderson, Houston, TX
MDA-T220	ATC	-	F	RPMI 1640, 10% FBS, 2mM L-Glu, 1 mM Na-Pyruvate, 50u/ml Pen/Strep	Dr. Stephen Lin, MD Anderson, Houston, TX	Dr. Stephen Lin, MD Anderson, Houston, TX
MDA-T245	ATC	-	F	RPMI 1640, 10% FBS, 2mM L-Glu, 1 mM Na-Pyruvate, 50u/ml Pen/Strep	Dr. Stephen Lin, MD Anderson, Houston, TX	Dr. Stephen Lin, MD Anderson, Houston, TX
MDA-T248	ATC	-	F	RPMI 1640, 10% FBS, 2mM L-Glu, 1 mM Na-Pyruvate, 50u/ml Pen/Strep	Dr. Stephen Lin, MD Anderson, Houston, TX	Dr. Stephen Lin, MD Anderson, Houston, TX
MDA-T269	ATC	59	M	RPMI 1640, 10% FBS, 2mM L-Glu, 1 mM Na-Pyruvate, 50u/ml Pen/Strep	Dr. Stephen Lin, MD Anderson, Houston, TX	Dr. Stephen Lin, MD Anderson, Houston, TX
MDA-T273	ATC	51	F	RPMI 1640, 10% FBS, 2mM L-Glu, 1 mM Na-Pyruvate, 50u/ml Pen/Strep	Dr. Stephen Lin, MD Anderson, Houston, TX	Dr. Stephen Lin, MD Anderson, Houston, TX
U-HTH7	ATC	74	F	MEM, 10% FBS, 1X NEAA, 2mM L-Glu, 50u/ml Pen/Strep	Dr. Stephen Lin, MD Anderson, Houston, TX	Dr. Nils Erik Heldin, Univ. of Uppsala, Sweden
U-HTH83	ATC	66	M	RPMI 1640, 10% FBS, 2mM L-Glu, 1 mM Na-Pyruvate, 50u/ml Pen/Strep	Dr. Stephen Lin, MD Anderson, Houston, TX	Dr. Nils Erik Heldin, Univ. of Uppsala, Sweden
U-HTH104	ATC	72	F	RPMI 1640, 10% FBS, 2mM L-Glu, 1 mM Na-Pyruvate, 50u/ml Pen/Strep	Dr. Stephen Lin, MD Anderson, Houston, TX	Dr. Nils Erik Heldin, Univ. of Uppsala, Sweden

(a): anaplastic thyroid cancer (ATC).

Supplemental Table S2. Cell line source and culture media (continued).

	Primary site[a]	Age	Sex	Culture media	Source	Primary source
CaSki	CIN	40	F	not in culture	in house	Dr. Richard F. Mattingly, Medical College of Wisconsin, Milwaukee, WI, USA
SiHa	CIN	55	F	not in culture	in house	Dr. Tohei Ito, Aichi Cancer Center, Nagoya, Japan
C-33 A	CIN	66	F	not in culture	in house	Dr. Nelly Auersperg, British Columbia Cancer Institute, Vancouver, British Columbia, Canada
HN30	HNSCC	-	M	DMEM, 10% FBS, 4.5 g/L D-Glucose, L-Glutamine, 50u/ml Pen/Strep	Dr. Vlad Sandulache, BCM, Houston, TX	Dr. Keith Robbins, NCI, NIH, Bethesda, MD
UM-SCC47	HNSCC	53	M	DMEM, 10% FBS, 4.5 g/L D-Glucose, L-Glutamine, 50u/ml Pen/Strep	Dr. Vlad Sandulache, BCM, Houston, TX	Dr. Thomas Carrey, Univ. of Michigan, Ann Arbor, MI
A375	MEL	54	F	DMEM, 10% FBS, 4.5 g/L D-Glucose, L-Glutamine, 50u/ml Pen/Strep	Dr. Ribes-Zamora, BCM, Houston, TX	Dr. Wade Parks, NCI, NIH, Bethesda, MD
HepG2	HCC	15	M	DMEM, 10% FBS, 4.5 g/L D-Glucose, L-Glutamine, 50u/ml Pen/Strep	Dr. Betty Slagle, BCM, Houston, TX	Dr. Barbara Knowles, Wistar Institute of Anatomy and Biology, Philadelphia, PA

(a): Cervical carcinoma (CIN), head and neck squamous cell carcinoma (HNSCC), melanoma (MEL), hepatocellular carcinoma (HCC).

Supplemental Table S3. MD Anderson STR profiling results.

Supplemental Table S4. Primer table.

Name	Amplicon	Sequence
RYR2.var1&2 _fw	937 bp surrounding rs10925366 and rs10802602 within *RYR2*	5' – CTCTCAGGCCTAAACCCAGT – 3'
RYR2.var1&2 _rv		5' – AGCTTTATTCATGAGGCCAGC – 3'
RYR_var1&2_seq_128fw	NA	5' – GCCTTCGGTTTGCTGTACTT – 3'
RYR_var 1&2_seq_103rv	NA	5' – ACATCTAGAGGGTAGGAATGCC – 3'
LRP1B_var_amp&seq_fw	1071 bp surrounding rs6702456 within *LRP1B*	5' – TGCATTTTATGAGTGAGTTCTGCT – 3'
LRP1B_var_amp_rv		5' – TGTGTTAGGAAACTTTTTGGAGCC – 3'
LRP1B_var_seq_130fw	NA	5' – GACATCCAAGCCTCCAGAAA – 3'
LRP1B_var _seq_138rv	NA	5' – GGTGCCTGTAATCCCAGCTA – 3'
FN1.var1&2_var_amp&seq_fw	940 bp surrounding rs10179937 and rs200077102 within *FN1*	5' – GGAACGGCATCAACTTGGAA – 3'
FN1.var1&2_var_amp&seq _rv		5' – GGCAAAAGCAAGACCGAGAG – 3'
FN1_var _seq_62rv	NA	5' – AGGGATGGCATTGTACCTGT – 3'
RADIL_MMD2_var_amp&seq_fw	896 bp surrounding rs13246949 upstream of *RADIL* and downstream of *MMD2*	5' – GCAGATTCACTAAGCCCATCTAA– 3'
RADIL_MMD2_var_amp&seq_rv		5' – TATGTGTAAGATGGACAGGGGTT– 3'
RADIL-MMD2_var_seq_238rv	NA	5' – GCGCTTCCAGAACATAGATAGA – 3'
CNTN5_var_amp&seq_fw	564 bp surrounding rs78588384 within *CNTN5*	5' – CCAACATCAAGTGGCCTTCT – 3'
CNTN5_amp&seq_var_rv		5' – AAGCAATCCTCCGACGTCTT – 3'
CNTN5_var_seq_118rv	NA	5' – CCCCTAATGTGGTTGCATTTGG – 3'
SERPINA1_var1&2_amp&seq _fw	1199 bp surrounding rs1987574 and rs78393784 downstream of *SERPINA1*	5' – ACCATGGGAAATTAGGTGAGACT – 3'
SERPINA1_var1&2_amp_rv		5' – CCCCAAAACAGCAGAAGACA – 3'
SERPINA1_var1&2_seq_rv	NA	5' – AATTATAGCCTGGGCATGG – 3'

Supplemental Table S5. PCR annealing and extension times and temperatures table.

Amplicon	Annealing	Extension
937 bp surrounding rs10925366 and rs10802602 within RYR2	30 seconds at 62.4°C	20 seconds at 72°C
1071 bp surrounding rs6702456 within LRP1B	30 seconds at 63.9°C	30 seconds at 72°C
940 bp surrounding rs10179937 and rs200077102 within FN1	30 seconds at 63.9°C	40 seconds at 72°C
896 bp surrounding rs13246949 upstream of RADIL and downstream of MMD2	30 seconds at 67.8°C	1 minute at 72°C
564 bp surrounding rs78588384 within CNTN5	30 seconds at 63.4°C	30 seconds at 72°C
1199 bp surrounding rs1987574 and rs78393784 downstream of SERPINA1	30 seconds at 63.6°C	40 seconds at 72°C

Supplemental Table S6. Cancer predisposition genes with papillary thyroid cancer expression data.

Supplemental Table S7. HERVs near cancer predisposition genes differentially expression in papillary thyroid cancer.

Supplemental Table S8. TGCA THCA patient metadata including demographic and clinical data.

Supplemental Table S9. 1KGP gender and ancestry data, training and validation subset assignment

Supplemental Table S10 Chromosomal locations of variants with significantly different frequencies in PTC tumor or PTC blood samples compared to 1KGP healthy controls.

Name	Chromosome	Hg19 Position	Hg38 Position	Location Relative to CPG	CPG
rs10802602	1	237,471,273	237,307,973	intron	RYR2
rs2618671	1	237,705,322	237,542,022	intron	RYR2
rs2779420	1	237,740,837	237,577,537	intron	RYR2
rs13030271	2	142,703,501	141,945,932	intron	LRP1B
rs10166768	2	142,865,187	142,107,618	intron	LRP1B
rs10179937	2	216,241,806	215,377,083	intron	FN1
rs200077102	2	216,241,808	215,377,085	intron	FN1
rs7682763	4	66,267,624	65,401,906	intron	EPHA5
rs13311049	7	4,179,047	4,139,415	intron	SDK1
rs13311637	7	4,179,137	4,139,505	intron	SDK1
rs611655	7	4,931,370	4,891,739	507 bp down (59,287 bp up)	MMD2 (RADIL)
rs12543616	8	92,956,746	91,944,518	14,112 bp down	RUNX1T1
rs10956571	8	132,013,792	131,001,546	intron	ADCY8
rs200093832	9	74,035,729	71,420,813	intron	TRPM3
rs61909780	11	100,138,014	100,267,282	intron	CNTN5
rs78588384	11	100,138,016	100,267,284	intron	CNTN5
rs1987574	14	94,825,633	94,359,296	17,531 bp down	SERPINA1
rs78393784	14	94,825,634	94,359,297	17,530 bp down	SERPINA1
rs370565365	16	20,463,884	20,452,562	intron	ACSM2A
rs112385920	19	6,581,068	6,581,057	3,483 bp down	CD70
rs2076859	21	36,792,234	35,419,936	intron	RUNX1
rs3989120	21	36,792,238	35,419,940	intron	RUNX1
rs13046555	21	36,899,230	35,526,932	intron	RUNX1
rs778825437	X	91,530,220	92,275,221	intron	PCDH11X
rs2754876	X	91,640,165	92,385,166	intron	PCDH11X
rs2750652	X	91,850,222	92,595,223	intron	PCDH11X

Supplemental Table S11. Rare variants with significantly different frequency in the TGCA data compared to gnomAD and non-detection in 1KGP

Supplemental Table S12. Minor allele frequencies of rare variants within Alu elements significantly different between PTC tumor or PTC blood samples and the Genome Aggregation Database (GnomAD).

SNP ID	Chr	BP hg38	CPG	MAF PTC Blood	MAF PTC Tumor	MAF GnomAD	*LD R^2	REF> ALT	Retro-element
rs111307085	1	69691822	LRRC7	0.12	0.17	0.04	0.7	G>A	Alu Jb poly(A)-tail
rs370175268	1	69691827	LRRC7	0.26	0.28	0.00003	0.7	G>*	Alu Jb poly(A)-tail
rs1397906922	1	237719083	RYR2	0.11	0.05	0.003	NA	A>C	Alu Jb
rs964437025	2	140629240	LRP1B	0.12	0.12	0.00001	0.91	G>T	Alu Sx4 linker
rs975889860	2	140629242	LRP1B	0.11	0.12	NA	0.91	A>T	Alu Sx4 linker
rs370725691	2	140673959	LRP1B	0.11	0.13	0.00003	<0.5	C>T	Alu Sx4 linker
rs750768534	3	135097116	EPHB1	0.13	0.11	0.001	NA	G>A	Alu Sc8 poly(A)-tail
rs376862741	7	88870410	ZNF804B	0.16	0.12	NA	0.6	T>G	Alu Yg6 poly(A)-tail
rs1372849291	7	88870414	ZNF804B	0.11	NA	NA	0.6	T>G	Alu Yg6 poly(A)-tail
rs200804978	8	15563706	TUSC3	0.14	0.14	0.00001	0.93	G>A	Alu Sx4 poly(A)-tail
rs368322211	8	15563709	TUSC3	0.13	0.14	0.00001	0.93	C>A	Alu Sx4 poly(A)-tail
rs765625195	9	70655331	TRPM3	0.28	0.25	NA	0.81	G>A	Alu Sg poly(A)-tail
rs1016132078	9	70655337	TRPM3	0.21	0.21	NA	0.81	G>A	Alu Sg poly(A)-tail
rs998328180	18	33564267	ASXL3	0.15	0.14	NA	NA	G>A	Alu Ya5 poly(A)-tail
rs1351688147	19	6602199	CD70	0.09	0.10	NA	NA	G>A	Alu Sq2 poly(A)-tail
rs13051966	21	34779885	LINC01426	0.97	0.97	NA	NA	C>*	Alu Jo poly(A)-tail
rs866722041	X	1230602	CRLF2	0.12	0.12	0.002	<0.5	T>G	Alu Sx1
rs867480422	X	1231833	CRLF2	0.10	0.20	0.00001	<0.5	T>G	Alu Sx1 poly(A)-tail
rs201048452	X	48250982	SSX1	0.17	0.14	0.0002	<0.5	C>*	Alu Sx1 poly(A)-tail

*Linkage disequilibrium (LD) was determined for variants in close proximity and expressed in terms of the squared correlation (R^2).

Supplemental Table S13. CPG expression in PTC from BioXpress and OncoMX.

Gene	Subjects Ratio	log2FC	FC	P-Value	Adjusted P-Value	Significance	Expression Trend
RYR2	56/59(94.92)	-1.06	-2.08	9.44E-30	2.74E-28	Yes	Down
LRP1B	58/59(98.31)	-1.21	-2.31	1.2E-21	1.94E-20	Yes	Down
FN1	54/59(91.53)	1.53	2.89	1.37E-108	1.84E-105	Yes	Up
RUNX1T1	56/59(94.92)	-1.02	-2.03	1.5E-24	3.02E-23	Yes	Down
TRPM3	51/59(86.44)	-1.17	-2.25	6.22E-09	3.05E-08	Yes	Down
CNTN5	56/59(94.92)	-1.35	-2.55	4.81E-26	1.08E-24	Yes	Down
SERPINA1	51/59(86.44)	1.57	2.97	3.24E-96	2.57E-93	Yes	Up
CD70	48/59(81.36)	1.17	2.25	1.18E-14	1.05E-13	Yes	Up
RUNX1	55/59(93.22)	1.35	2.55	1.1E-54	1.28E-52	Yes	Up
PCDH11X	57/59(96.61)	-1.15	-2.22	1.63E-19	2.23E-18	Yes	Down

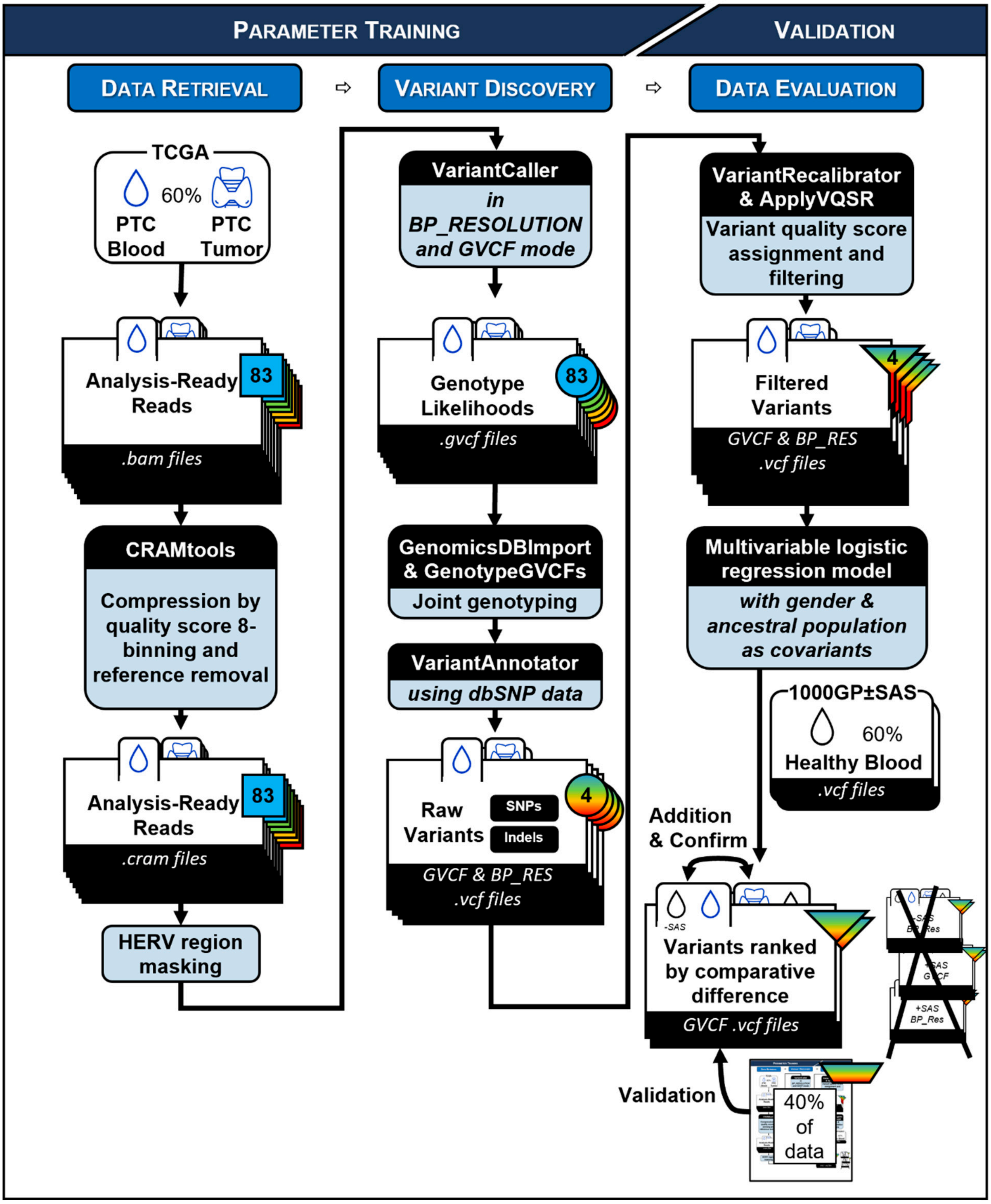
PARAMETER TRAINING
VALIDATION
DATA RETRIEVAL
VARIANT DISCOVERY
DATA EVALUATION
TCGA
60%
PTC Blood
PTC Tumor
Analysis-Ready Reads
83
.bam files
CRAMtools
Compression by quality score 8-binning and reference removal
Analysis-Ready Reads
83
.cram files
HERV region masking
VariantCaller
in BP_RESOLUTION and GVCF mode
Genotype Likelihoods
83
.gvcf files
GenomicsDBImport & GenotypeGVCFs
Joint genotyping
VariantAnnotator
using dbSNP data
Raw Variants
SNPs
Indels
4
GVCF & BP_RES .vcf files
VariantRecalibrator & ApplyVQSR
Variant quality score assignment and filtering
Filtered Variants
4
GVCF & BP_RES .vcf files
Multivariable logistic regression model
with gender & ancestral population as covariants
1000GP±SAS
60%
Healthy Blood
.vcf files
Addition & Confirm
-SAS
Variants ranked by comparative difference
GVCF .vcf files
Validation
40% of data

Supplemental Figure S1. Variant calling pipeline scheme. This pipeline was used to detect single nucleotide variants (SNV) and small insertion/deletions (indels) in human endogenous retroviral (HERV) sequences within the Papillary Thyroid Cancer (PTC) dataset from The Cancer Genome Atlas (TCGA). For storage, BAM (binary alignment map) files were converted into a 3-5 times smaller CRAM (compressed reference-oriented alignment map) files. Detected variants from the TCGA dataset were compared with variants detected in HERV regions from the 1000 genomes project (1KGP) without South-East Asian samples. Statistically different variants detected in 60% of the samples were validated in the remaining 40% by comparing PTC blood and tumor samples to the 1KGP dataset without the SAS superpopulation sample using VariantCaller in GVCF mode. File extensions are indicated in white font on black background at the bottom of each box while GATK or statistical tools are denoted in white font on black background at the top. Number in solid boxes signify the number of individuals evaluated, the number on multi-color background indicate the number of different files.

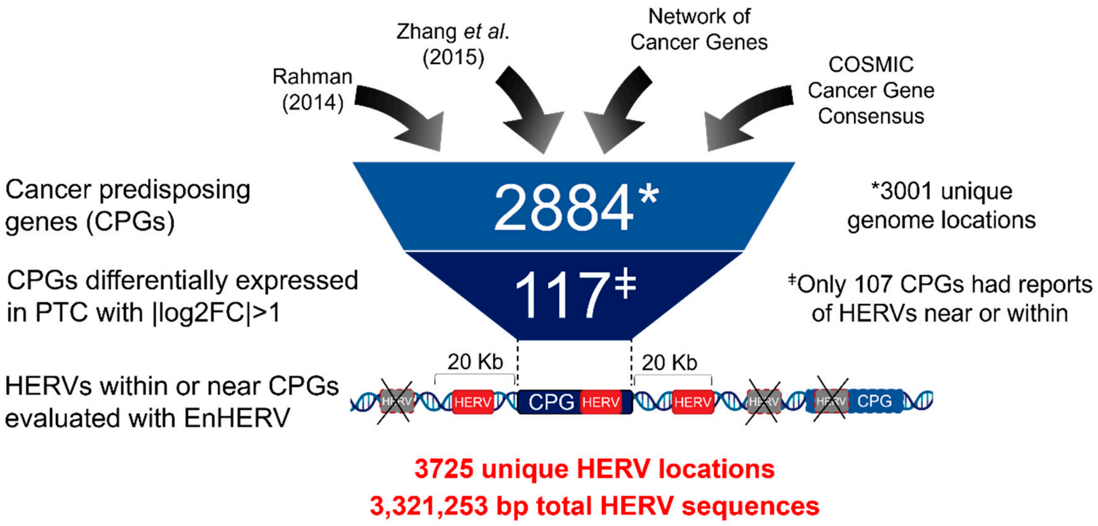

Supplemental Figure S2. Extraction of HERVs within 20 Kbp radius of differentially expressed CPGs in PTC. CPGs were obtained from Rahman (2014) [63], a primary research article by Zhang *et al.* (2015) [64], the Network of Cancer Genes [65], and the COSMIC Cancer Gene Consensus [66]. Differential expression of CPGs was determined using BioXpress TCGA PTC mRNA data. All genes with more or less than 2-fold change (1 log2FC) were evaluated for HERV sequences in proximity using the EnHERV database [88].

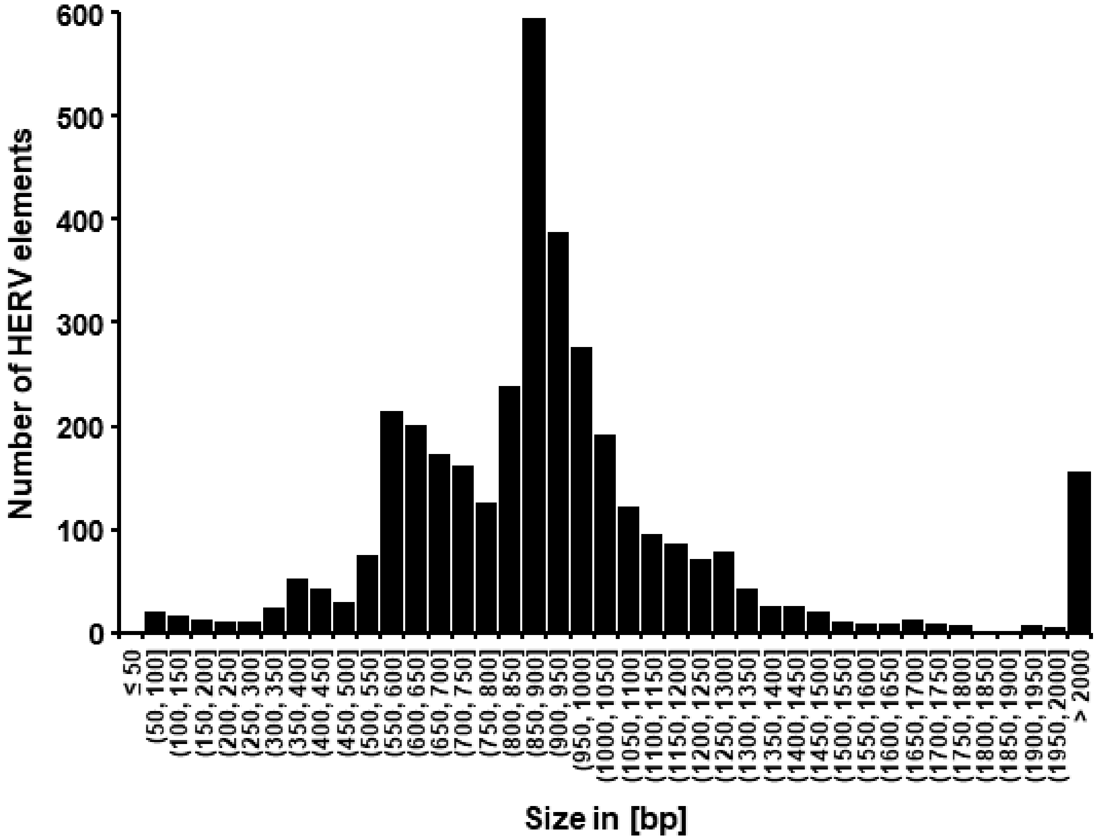

Supplemental Figure S3. Size distribution of HERVs near of within CPGs. A total of 3725 HERV sequences are located within 20 Kbp distance or inside of CPGs regions. The HERV sequences had a median size of 882 bp (25% quartile = 707 bp, 75% quartile = 1022 bp).

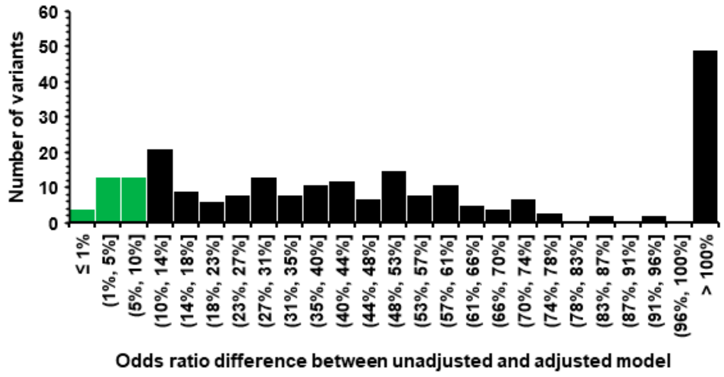

(a)

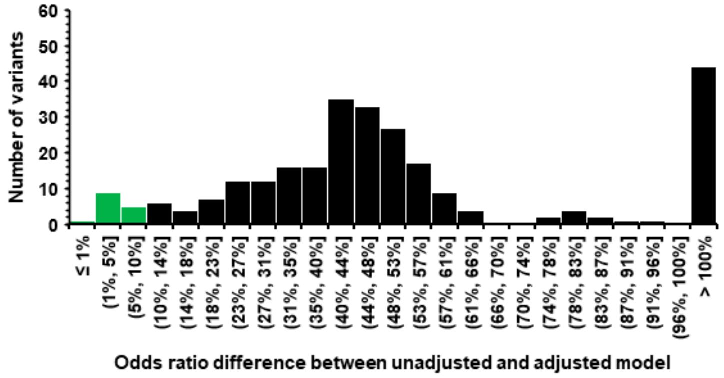

(b)

Supplemental Figure S4. Distribution of odds ratio (OR) changes for variants with significant p-values from the training set. OR differences were calculated by dividing the absolute difference of ORs from the unadjusted logistic regression analyses and multivariate regression analyses by the OR of the multivariate regression analyses for (a) PTC blood samples compared to healthy controls and (b) PTC tumor samples compared to healthy controls. Variants unaffected by covariates defined as OR difference ≤10% are marked in green.

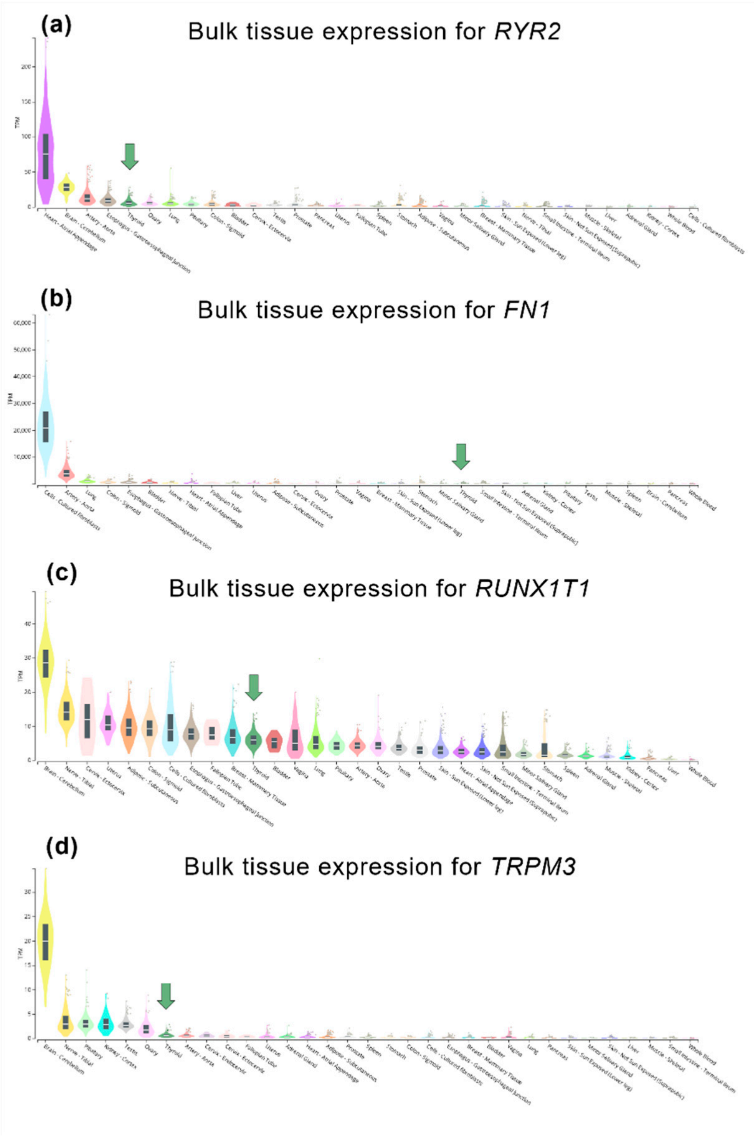
(a)
Bulk tissue expression for *RYR2*
(b)
Bulk tissue expression for *FN1*
(c)
Bulk tissue expression for *RUNX1T1*
(d)
Bulk tissue expression for *TRPM3*

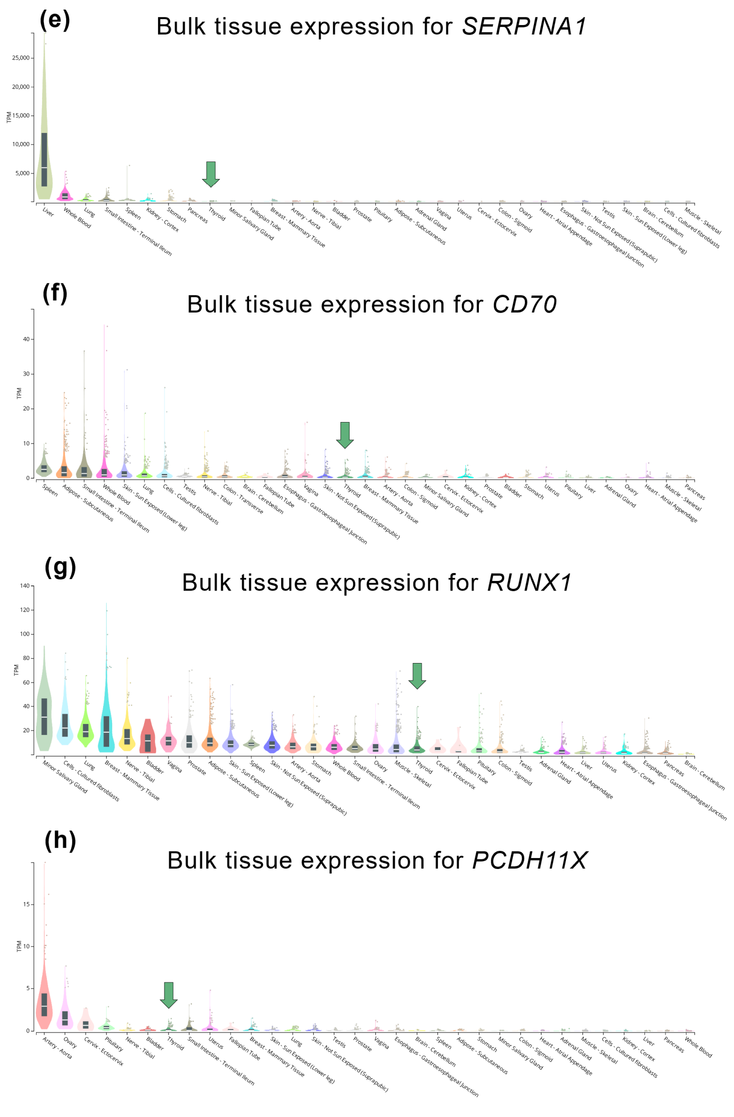

Supplemental Figure S5. RYR2, FN1, RUNX1T1, SERPINA1, CD70, RUNX1 and PCDH11X mRNA expression in patients with PTC and normal tissues. The mRNA levels in healthy tissues presented in TPM (transcripts per million) were derived from the Genotype-Tissue Expression (GTEx) portal [14]. The sum of all TPM values is similar in all samples, so that a TPM value signifies a relative expression level, in principle, allowing the comparison between samples [96].

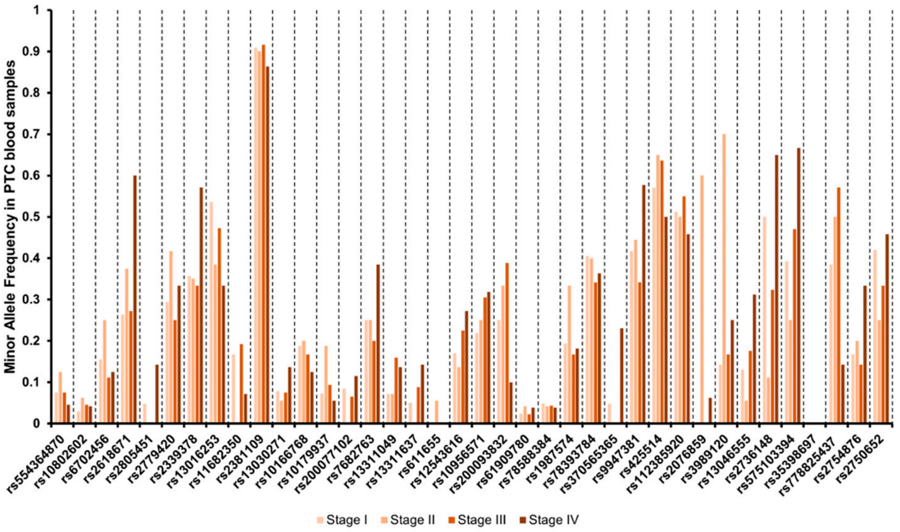

(a)

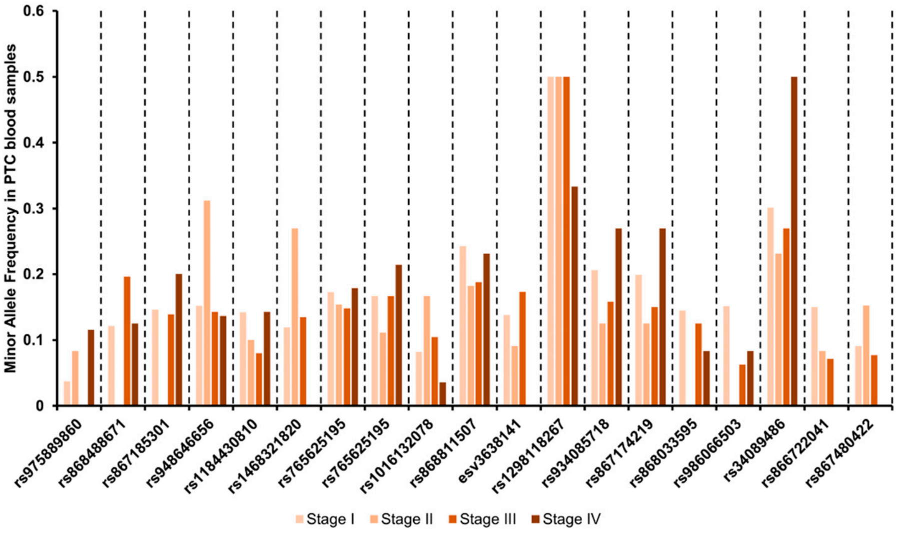

(b)

Supplemental Figure S6. Minor allele frequencies of different variants in TGCA PTC tumor samples according to stage.

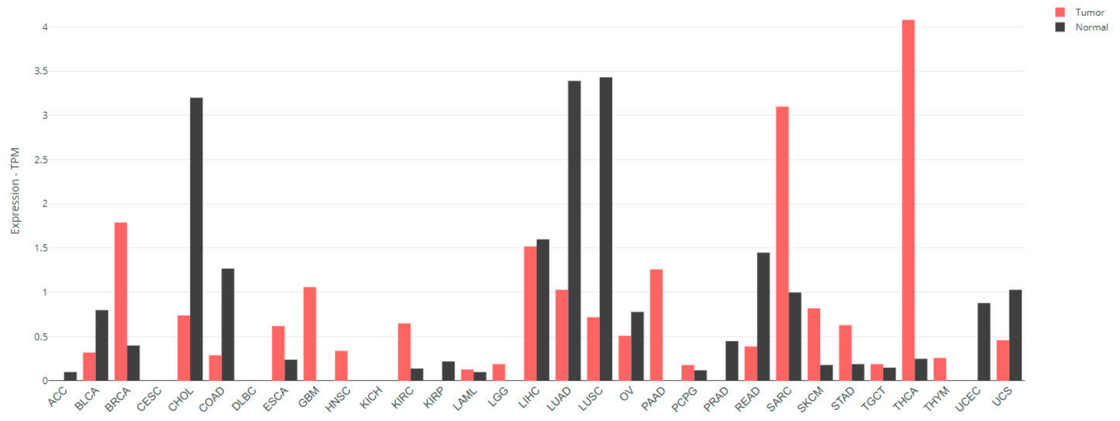
Tumor
Normal
Expression - TPM
ACC BLCA BRCA CESC CHOL COAD DLBC ESCA GBM HNSC KICH KIRC KIRP LAML LGG LIHC LUAD LUSC OV PAAD PCPG PRAD READ SARC SKCM STAD TGCT THCA THYM UCEC UCS

(a)

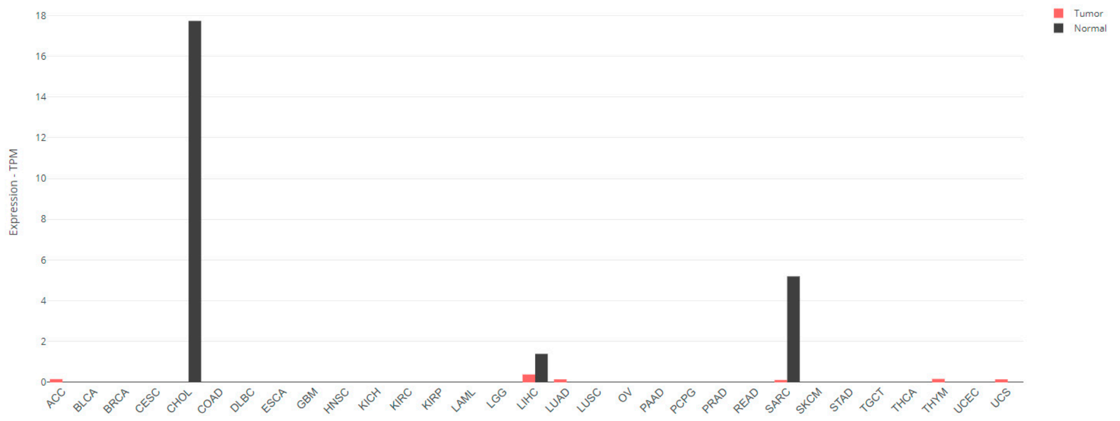
Tumor
Normal
Expression - TPM
ACC BLCA BRCA CESC CHOL COAD DLBC ESCA GBM HNSC KICH KIRC KIRP LAML LGG LIHC LUAD LUSC OV PAAD PCPG PRAD READ SARC SKCM STAD TGCT THCA THYM UCEC UCS

(b)

Supplemental Figure S7. Expression profiles of different *FN1* isoforms. Expression of *FN1* alternative transcripts (a) ENST00000460217.1, and (b) ENST00000438981.1 in different TCGA cancers and normal controls. TCGA tumor type abbreviation codes are as follows: ACC, adrenocortical carcinoma; BLCA, bladder urothelial carcinoma; BRCA, breast invasive carcinoma; CESC, cervical squamous cell carcinoma and endocervical adenocarcinoma; CHOL, cholangiocarcinoma; COAD, colon adenocarcinoma; DLBC, diffuse large B-cell lymphoma; ESCA, oesophageal carcinoma; GBM, glioblastoma multiforme; HNSC, head and neck squamous cell carcinoma; KICH, chromophobe renal cell carcinoma; KIRC, clear-cell renal clear cell carcinoma; KIRP, papillary renal cell carcinoma; LAML, acute myeloid leukemia; LGG, lower-grade glioma; LIHC, hepatocellular carcinoma; LUAD, lung adenocarcinoma; LUSC, lung squamous cell carcinoma; MESO, mesothelioma; OV, ovarian serous adenocarcinoma; PAAD, pancreatic adenocarcinoma; PCPG, phaeochromocytoma and paraganglioma; PRAD, prostate adenocarcinoma; READ, rectal adenocarcinoma; SARC, adult soft-tissue sarcoma; SKCM, cutaneous melanoma; STAD, stomach adenocarcinoma; TGCT, testicular germ cell tumor; THCA, thyroid carcinoma; THYM, thymoma; UCEC, uterine corpus endometrial carcinoma; UCS, uterine carcinosarcoma; UVM, uveal melanoma.

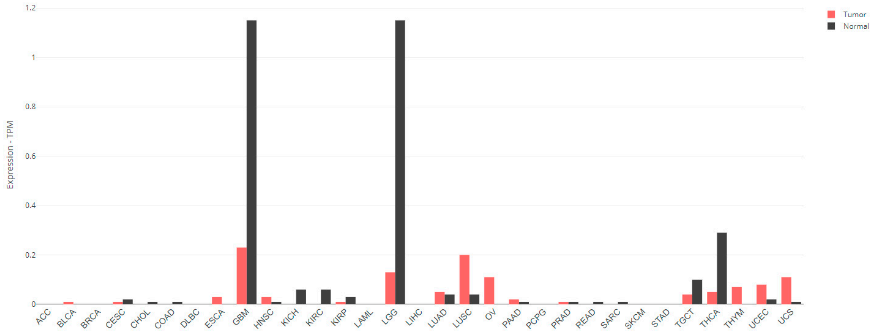
Tumor
Normal
Expression - TPM
1.2
1
0.8
0.6
0.4
0.2
0
ACC
BLCA
BRCA
CESC
CHOL
COAD
DLBC
ESCA
GBM
HNSC
KICH
KIRC
KIRP
LAML
LGG
LIHC
LUAD
LUSC
OV
PAAD
PCPG
PRAD
READ
SARC
SKCM
STAD
TGCT
THCA
THYM
UCEC
UCS

(a)

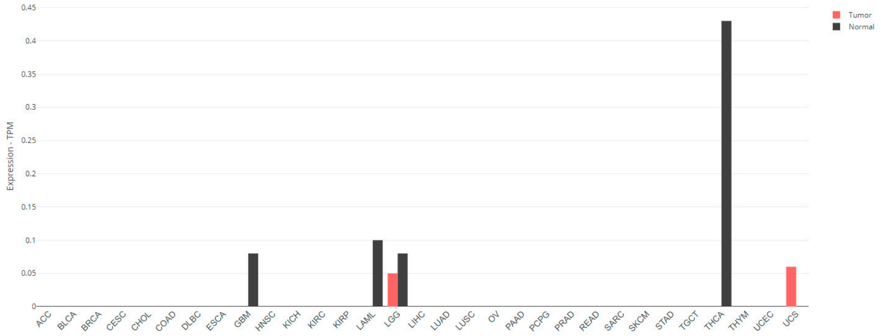
Tumor
Normal
Expression - TPM
0.45
0.4
0.35
0.3
0.25
0.2
0.15
0.1
0.05
0
ACC
BLCA
BRCA
CESC
CHOL
COAD
DLBC
ESCA
GBM
HNSC
KICH
KIRC
KIRP
LAML
LGG
LIHC
LUAD
LUSC
OV
PAAD
PCPG
PRAD
READ
SARC
SKCM
STAD
TGCT
THCA
THYM
UCEC
UCS

(b)

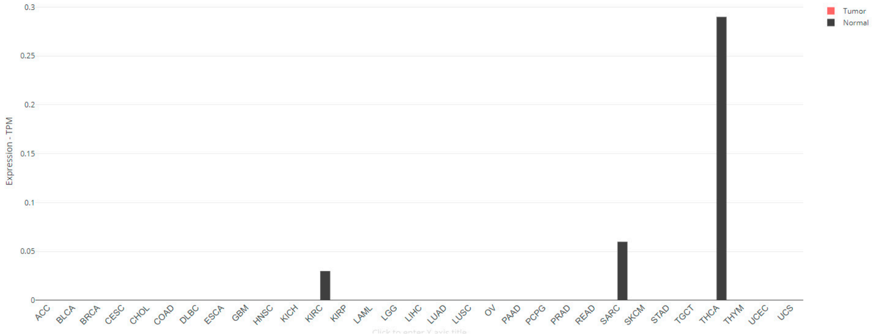
Tumor
Normal
Expression - TPM
0.3
0.25
0.2
0.15
0.1
0.05
0
ACC
BLCA
BRCA
CESC
CHOL
COAD
DLBC
ESCA
GBM
HNSC
KICH
KIRC
KIRP
LAML
LGG
LIHC
LUAD
LUSC
OV
PAAD
PCPG
PRAD
READ
SARC
SKCM
STAD
TGCT
THCA
THYM
UCEC
UCS

(c)

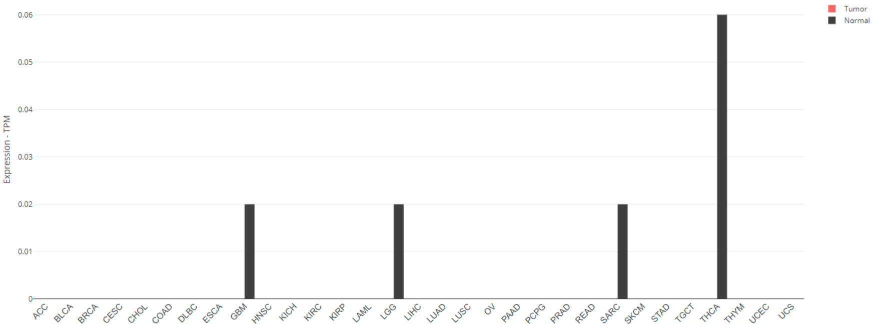
Tumor
Normal
Expression - TPM
0.06
0.05
0.04
0.03
0.02
0.01
0
ACC
BLCA
BRCA
CESC
CHOL
COAD
DLBC
ESCA
GBM
HNSC
KICH
KIRC
KIRP
LAML
LGG
LIHC
LUAD
LUSC
OV
PAAD
PCPG
PRAD
READ
SARC
SKCM
STAD
TGCT
THCA
THYM
UCEC
UCS

(d)

Supplemental Figure S8. Expression profiles of different *CNTN5* isoforms. Expression of *CNTN5* alternative transcripts (a) ENST00000619298, (b) ENST00000525047.1, (c) ENST00000524871.5, and (d) ENST00000528727.5 in different TCGA cancers and normal controls. TCGA tumor type abbreviation codes are as follows: ACC, adrenocortical carcinoma; BLCA, bladder urothelial carcinoma; BRCA, breast invasive carcinoma; CESC, cervical squamous cell carcinoma and endocervical adenocarcinoma; CHOL, cholangiocarcinoma; COAD, colon adenocarcinoma; DLBC, diffuse large B-cell lymphoma; ESCA, oesophageal carcinoma; GBM, glioblastoma multiforme; HNSC, head and neck squamous cell carcinoma; KICH, chromophobe renal cell carcinoma; KIRC, clear-cell renal clear cell carcinoma; KIRP, papillary renal cell carcinoma; LAML, acute myeloid leukemia; LGG, lower-grade glioma; LIHC, hepatocellular carcinoma; LUAD, lung adenocarcinoma; LUSC, lung squamous cell carcinoma; MESO, mesothelioma; OV, ovarian serous adenocarcinoma; PAAD, pancreatic adenocarcinoma; PCPG, phaeochromocytoma and paraganglioma; PRAD, prostate adenocarcinoma; READ, rectal adenocarcinoma; SARC, adult soft-tissue sarcoma; SKCM, cutaneous melanoma; STAD, stomach adenocarcinoma; TGCT, testicular germ cell tumor; THCA, thyroid carcinoma; THYM, thymoma; UCEC, uterine corpus endometrial carcinoma; UCS, uterine carcinosarcoma; UVM, uveal melanoma.
