# Supplementary material for: Targeted Variant Assessments of Human Endogenous Retroviral Regions in Whole Genome Sequencing Data Reveal Retroviral Variants Associated with Papillary Thyroid Cancer
Source: Microorganisms. 2024 Nov 27;12(12):2435. doi: 10.3390/microorganisms12122435 (PMC11679660; doi:10.3390/microorganisms12122435)
Supplement: Supplementary file 1 [file microorganisms-12-02435-s001.zip › Supplemental Table S3. STR sequencing results MD Anderson.pdf]

THE UNIVERSITY OF TEXAS
MD Anderson Cancer Center
Making Cancer History®

Cytogenetics and Cell Authentication Core (CCAC)

https://www.mdanderson.org/research/research-resources/core-facilities/cytogenetics-and-cell-authentication-core.html

CLC
Cell Line Characterization

SET659	4/10/2023

Expiration date: 12/31/2023

Source	Sample Name	AMEL	CSF1PO	D13S317	D16S539	D18S51	D21S11	D3S1358	D5S818	D7S820	D8S1179	FGA	TH01	TPOX	vWA	Comments
Erik Stricker	CaSki	X	10	8,12	11,12	17	30	15	13	8,11	15	21	7	8	17	
Public database GenenTech	CA-Ski	X	10	8, 12	11, 12	17	30	15	13	8, 11	15	21	7	8	17	
Public database Sanger	CA-SKI	X	10	8,12	11,12	17	30	15	13	8,11	15	21	7	8	17	MATCH

Source	Sample Name	AMEL	CSF1PO	D13S317	D16S539	D18S51	D21S11	D3S1358	D5S818	D7S820	D8S1179	FGA	TH01	TPOX	vWA	Comments
Erik Stricker	SiHa	X	12	11	12	15	29,31	16,17	9	10	13,16	21	6,9	8	14,17	
DSMZ,ATCC,JCRB,RIKEN online search database	SIHA	X	12	11	12	15,16	29,31	16,17	9	10	13,16	21	6,9	8	14,17	MATCH

Source	Sample Name	AMEL	CSF1PO	D13S317	D16S539	D18S51	D21S11	D3S1358	D5S818	D7S820	D8S1179	FGA	TH01	TPOX	vWA	Comments
Erik Stricker	UMSCC 47	X,Y	11,13	8,11	8,13	18	29,30	15	11,12	11	15	25	7,9.3	10,11	18	
Clinical Cancer Research	UM-SCC-47	X,Y	11,13	8,11	8,13	18	29,30	15	11,12	11	15	25	7,9.3	10,11	18	
Head Neck 2010 April 32(4): 417-426	UM-SCC-47	X,Y		8,11		18	29,30	15	11,12	11	15	23,25			18	MATCH

Source	Sample Name	AMEL	CSF1PO	D13S317	D16S539	D18S51	D21S11	D3S1358	D5S818	D7S820	D8S1179	FGA	TH01	TPOX	vWA	Comments
Erik Stricker	HN30 2	X,Y	10,12	11,12	11,12	13,16	28	16	12,13	10	15,17	20,24	7,9	6,11	15,18	
CCLC customer database	HN30	X,Y	10,12	11,12	11,12	13,16	28	16	12,13	10	15,17	20,24	7,9	6,11	15,18	MATCH

Source	Sample Name	AMEL	CSF1PO	D13S317	D16S539	D18S51	D21S11	D3S1358	D5S818	D7S820	D8S1179	FGA	TH01	TPOX	vWA	Comments
Erik Stricker	HN5	X,Y	9,10,12	9,10,11,12	10,11,12	13,15,16	28,31.2,32	13,16	9,12,13	10,11	13,15,17	20,21,24,2[illegible]	6,7,9,9.3	6,8,11	15,17,18	*Mixed cell line profile*
Clinical Cancer Research	HN5	X	11,13	11	11,13	13,15	28,31.2	18	13	11	11,12	24	9.3	8,11	15,18,19	

Source	Sample Name	AMEL	CSF1PO	D13S317	D16S539	D18S51	D21S11	D3S1358	D5S818	D7S820	D8S1179	FGA	TH01	TPOX	vWA	Comments
Erik Stricker	A375	X	11,12	11,14	9	12,17	29,30	15,17	12	9	11,14	20,23	8	8,10	16,17	
DSMZ, ATCC ,JCRB ,RIKEN online search database	A-375	X	11,12	11,14	9	17	29,30	15,17	12	9	11,14	20,23	8	8,10	16,17	MATCH

Source	Sample Name	AMEL	CSF1PO	D13S317	D16S539	D18S51	D21S11	D3S1358	D5S818	D7S820	D8S1179	FGA	TH01	TPOX	vWA	Comments
Erik Stricker	HepG2	X,Y	10,11	9,13	12,13	13,14	29,31	15,16	11,12	10	15,16	22,25	9	8,9	17	
Public database CLS	HEP-G2	X,Y	10,11	9,13	12,13	13,14	29,31	15,16	11,13	10	15,16,17	22,26	9	8,9	17	MATCH

Source	Sample Name	AMEL	CSF1PO	D13S317	D16S539	D18S51	D21S11	D3S1358	D5S818	D7S820	D8S1179	FGA	TH01	TPOX	vWA	Comments
Erik Stricker	HeLa	X	12	9	10,11	15,16	29	15	11,12	8,11	13,14	19,24	6	8	20	
Public database GenenTech	HeLA	X	9, 10	12, 13.3	9, 10	16	27, 28	15, 18	11, 12	8, 12	12, 13	18, 21	7	8, 12	16, 18	Profile does not match Hela

Source	Sample Name	AMEL	CSF1PO	D13S317	D16S539	D18S51	D21S11	D3S1358	D5S818	D7S820	D8S1179	FGA	TH01	TPOX	vWA	Comments
Erik Stricker	HN31 1 24 22 1	X,Y	10,12	11,12	11,12	13,16	28	16	12,13	10	15,17	20,24	7,9	6,11	15,18	
Clinical Cancer Research	HN31	X,Y	10,12	11,12	11,12	13,16	28	16	12,13	10	15,17	20,24	7,9	6,11	15,18	MATCH

Source	Sample Name	AMEL	CSF1PO	D13S317	D16S539	D18S51	D21S11	D3S1358	D5S818	D7S820	D8S1179	FGA	TH01	TPOX	vWA	Comments
Erik Stricker	C33A	X	12	13	13,14	15,18	29,30,31	16	11,12	10	10,14	21,26	7,8	9	18,20	
Public database Sanger	C33A	X	12	13	13,14	15,18	29,30	16	11,12	10	10,14	21,26	7,8	9	18,20	MATCH

Source	Sample Name	AMEL	CSF1PO	D13S317	D16S539	D18S51	D21S11	D3S1358	D5S818	D7S820	D8S1179	FGA	TH01	TPOX	vWA	Comments
Erik Stricker	U HTH83	X,Y	11	11,13	11,12	12,14	28,32.2	17	12	12	13	23	6,9	8	16,19	
Clinical Cancer Research	U-Hth83	X,Y	11	11,13	11,12	12,14	28,32.2	17	12	12	13,17	23	6,9	8	16,19	MATCH

Source	Sample Name	AMEL	CSF1PO	D13S317	D16S539	D18S51	D21S11	D3S1358	D5S818	D7S820	D8S1179	FGA	TH01	TPOX	vWA	Comments
Erik Stricker	U HTH7	X	12	11,14	9,13	16	29,30	14,15	11	8,11	12,13	20,23	9,9.3	11	14,18	
Clinical Cancer Research	U-Hth7	X	12	11,14	9,13	16	29,30	14,15	11	8,11	12,13	20,23	9,9.3	11	14,18	MATCH

Source	Sample Name	AMEL	CSF1PO	D13S317	D16S539	D18S51	D21S11	D3S1358	D5S818	D7S820	D8S1179	FGA	TH01	TPOX	vWA	Comments
Erik Stricker	U HTH104	X	12	12	9,12	14,19	27,31.2	14	12	10	12,13	23	6,9.3	8,11	14,16	
Clinical Cancer Research	U-Hth104	X	12	12	9,12	14,19	27,31.2	14	12	10	12,13	23	6,9.3	8,11	14,16	MATCH

Source	Sample Name	AMEL	CSF1PO	D13S317	D16S539	D18S51	D21S11	D3S1358	D5S818	D7S820	D8S1179	FGA	TH01	TPOX	vWA	Comments
Erik Stricker	TPC 1	X	11,12	11,12	9	13,16	30,31.2	16,17	8,10	11	11,17	20,21	9	11	14,18	
CCLC customer database	TPC1	X	11,12	11,12	9	16	30,31.2	16,17	8,10	11	11,17	21	9	11	14	MATCH

Source	Sample Name	AMEL	CSF1PO	D13S317	D16S539	D18S51	D21S11	D3S1358	D5S818	D7S820	D8S1179	FGA	TH01	TPOX	vWA	Comments
Erik Stricker	MDA T220	X	9,10	12	10,11	11,14	33.2,34.2	16,17	13	8,11	13,14	25	6,9	9,11	15,16	
CCLC customer database set 378	MDA-T220	X	9,10	12	10,11	11,14	33.2,34.2	16,17	13	8,11	13,14	25	6,9	9,11	15,16	MATCH

Source	Sample Name	AMEL	CSF1PO	D13S317	D16S539	D18S51	D21S11	D3S1358	D5S818	D7S820	D8S1179	FGA	TH01	TPOX	vWA	Comments
Erik Stricker	MDA T120	X	12,14	11	13	16	30,32.2	15,16	11,12	10,11	13	19,23	6,9	8,9	14,17	
CCLC customer database- SET 193-(Gary Clayman)	P120	X	12,14	11	13	15,16	30,32.2	15,16	11,12	10,11	13	19,23	6,9	8,9	14,17	MATCH

Source	Sample Name	AMEL	CSF1PO	D13S317	D16S539	D18S51	D21S11	D3S1358	D5S818	D7S820	D8S1179	FGA	TH01	TPOX	vWA	Comments
Erik Stricker	MDA T248	X	12	11	11,13	12,14	30,31	14	9,11	11	11,13	25	6,8	9,10	14,18	
CCLC customer database SET 492	p248	X	12	11	11,13	12,14	30,31	14	9,11	11	11,13	25	6,8	9,10	14,18	MATCH

Source	Sample Name	AMEL	CSF1PO	D13S317	D16S539	D18S51	D21S11	D3S1358	D5S818	D7S820	D8S1179	FGA	TH01	TPOX	vWA	Comments
Erik Stricker	MDA T178	X	10,12	8,9	12,13	12,16	30,31.2	15,16	9,13	9,10	12,15	20,21	7	8,11	17,18	
CCLC customer database SET 483	MDA-T178	X	10,12	8,9	12,13	12,16	30,31.2	15,16	9,13	9,10	12,15	20,21	7	8,11	17,18	MATCH

Source	Sample Name	AMEL	CSF1PO	D13S317	D16S539	D18S51	D21S11	D3S1358	D5S818	D7S820	D8S1179	FGA	TH01	TPOX	vWA	Comments
Erik Stricker	MDA T85	X,Y	9,10	9,10	10	13,15	31.2,32.2	13,16	9,12	11	13	21,27	6,9.3	8	17	
CCLC customer database- SET 193-(Gary Clayman)	P85	X,Y	9,10	9,10	10	13,15	31.2,32.2	13,16	9,12	11	13	21,27	6,9.3	8	17	MATCH

Source	Sample Name	AMEL	CSF1PO	D13S317	D16S539	D18S51	D21S11	D3S1358	D5S818	D7S820	D8S1179	FGA	TH01	TPOX	vWA	Comments
Erik Stricker	MDA T189	X	11,12	10	11	15	31.2,32.2	17	11,13	11	13	19,23	7,9	8	16,17	
CCLC customer database - SET 337-(Gary Clayman)	P189	X	11,12	10,11	11	13,15	31.2	15,17	11,13	11	13	19,23	7,9	8	16,17	MATCH

Source	Sample Name	AMEL	CSF1PO	D13S317	D16S539	D18S51	D21S11	D3S1358	D5S818	D7S820	D8S1179	FGA	TH01	TPOX	vWA	Comments
Erik Stricker	MDA T245	X	10,12	11	12	13,19	29,32.2	15	13,14	11	12	22,26	7,9.3	8,11	16,17	
CCLC customer database SET433	P245	X	10,12	11	11,12	13,19	29,32.2	15	13,14	11	12	22,26	7,9.3	8,11	16,17	MATCH

Source	Sample Name	AMEL	CSF1PO	D13S317	D16S539	D18S51	D21S11	D3S1358	D5S818	D7S820	D8S1179	FGA	TH01	TPOX	vWA	Comments
Erik Stricker	MDA T273	X	11,12	9	11	15,16	30,31	18	12	10	15	19,25	7,9	8,11	14	
CCLC customer database SET 492	p273	X	11,12	9	11	15,16	30,31	18	12	10,11	15	19,25	7,9	8,11	14	MATCH

Source	Sample Name	AMEL	CSF1PO	D13S317	D16S539	D18S51	D21S11	D3S1358	D5S818	D7S820	D8S1179	FGA	TH01	TPOX	vWA	Comments
Erik Stricker	MDA T171	X	10,11	12	11,12	13,15	29,30	15,16	11,12	10,11	11,14	21,23	6,7	9,11	14,18	
CCLC customer database -SET 324 (MD Anderson Lab)	YH14	X	10,11	12	11,12	13,15	29,30	15,16	11,12	10,11	11,14	21,23	6,7	9,11	14,18	MATCH

Source	Sample Name	AMEL	CSF1PO	D13S317	D16S539	D18S51	D21S11	D3S1358	D5S818	D7S820	D8S1179	FGA	TH01	TPOX	vWA	Comments
Erik Stricker	MDA T269	X	11,14	11,12	11,13	13,16	29,32.2	17	12	10	13	18,26	6	8,11	14,17	
CCLC customer database SET 492	p269	X,Y	11,14	11,12	11,13	13,16	29,32.2	17	12	10	13	18,26	6	8,11	14,17	MATCH

Source	Sample Name	AMEL	CSF1PO	D13S317	D16S539	D18S51	D21S11	D3S1358	D5S818	D7S820	D8S1179	FGA	TH01	TPOX	vWA	Comments
Erik Stricker	MDA T187	X	12	12	11	17,19	31.2,32.2	14,16	14	9,12	14,15	23,24	9.3	8	16,18	
CCLC customer database -MD-Anderson Cell line	MDA-T187	X	10,12	12	11	17,19	31.2,32.2	14,16	11,14	12	14,15	23,24	9.3	8	16,18	MATCH

Source	Sample Name	AMEL	CSF1PO	D13S317	D16S539	D18S51	D21S11	D3S1358	D5S818	D7S820	D8S1179	FGA	TH01	TPOX	vWA	Comments
Erik Stricker	MDA T192	X	11,13	11	9,11	17	27,28	17	11,12	8,12	13	20,24	6,9	8,11	17	
CCLC customer database -SET 364-(Gary Clayman)	P192	X	11,13	11,13	9,11	17	27,28	17	11,12	8,12	13	20,24	6,9	8,11	17	MATCH

Source	Sample Name	AMEL	CSF1PO	D13S317	D16S539	D18S51	D21S11	D3S1358	D5S818	D7S820	D8S1179	FGA	TH01	TPOX	vWA	Comments
Erik Stricker	MDA T22	X	11,12	11,14	9,11	13,19	28,30	15,17	11,13	9,13	12,14	22	6,9	8,10	14,18	Profile unique no match found
Erik Stricker	MDA T41	X,Y	11,12	9,12	12	12,14	29,33.2	15,17	9,12	8,12	12,13	21,23	6	8	15,16	Profile unique no match found
Erik Stricker	MDA T68	X,Y	11	9,12	11,12	15,18	30	16,18	12	9,12	14,16	23	7	8,12	18	Profile unique no match found
Erik Stricker	MDA T32	X	11,12	12	10,12	13	29,30,31	15,17	11,12	10	12	20,26	6,9.3	8,11	16,17	Profile unique no match found

CCAC database is a comprehensive databases of short tandem repeat (STR) profiles, The database includes profiles from 20 public database/ publications and the cell lines developed by MD Anderson research labs.
The database contains over 5000 unique human cancer cell line STR profiles, one of the largest cancer cell line STR online search database in the world

Annual cell line authentication is required by UTMDACC INSTITUTIONAL POLICY # ACA1044

THE UNIVERSITY OF TEXAS
MD Anderson ~~Cancer~~ Center
Making Cancer History®

Cytogenetics and Cell Authentication Core (CCAC)

https://www.mdanderson.org/research/research-resources/core-facilities/cytogenetics-and-cell-authentication-core.html

CLC
Cell Line Characterization

SET 603	2/25/2022

Expiration date: 9/30/2022

Source	Sample_Name	AMEL	CSF1PO	D13S317	D16S539	D18S51	D21S11	D3S1358	D5S818	D7S820	D8S1179	FGA	TH01	TPOX	vWA	Comments
Defeng Deng	HN30	X,Y	10,12	11,12	11,12	13,16	28	16	12,13	10	15,17	20,24	7,9	6,11	15,18	
Defeng Deng	HN30e1	X,Y	10,12	11,12	11,12	13,16	28	16	12,13	10	15,17	20,24	7,9	6,11	15,18	
CCLC customer database	HN30	X,Y	10,12	11,12	11,12	13,16	28	16	12,13	10	15,17	20,24	7,9	6,11	15,18	MATCH

Source	Sample_Name	AMEL	CSF1PO	D13S317	D16S539	D18S51	D21S11	D3S1358	D5S818	D7S820	D8S1179	FGA	TH01	TPOX	vWA	Comments
Defeng Deng	HN31	X,Y	10,12	11,12	11,12	13,16	28	16	12,13	10	15,17	20,24	7,9	6,11	15,18	
Defeng Deng	HN31P10A	X,Y	10,12	11,12	11,12	13,16	28	16	12,13	10	15,17	20,24	7,9	6,11	15,18	
Defeng Deng	HN31P10C	X,Y	10,12	11,12	11,12	13,16	28	16	12,13	10	15,17	20,24	7,9	6,11	15,18	
Clinical Cancer Research	HN31	X,Y	10,12	11,12	11,12	13,16	28	16	12,13	10	15,17	20,24	7,9	6,11	15,18	MATCH

Source	Sample_Name	AMEL	CSF1PO	D13S317	D16S539	D18S51	D21S11	D3S1358	D5S818	D7S820	D8S1179	FGA	TH01	TPOX	vWA	Comments
Defeng Deng	HN5	X,Y	10,12	11,12	11,12	13,16	28	16	12,13	10	15,17	20,24	7,9	6,11	15,18	
Clinical Cancer Research	HN5	X	11,13	11	11,13	13,15	28,31.2	18	13	11	11,12	24	9.3	8,11	15,18,19	Profile does not match HN5
Clinical Cancer Research	HN31	X,Y	10,12	11,12	11,12	13,16	28	16	12,13	10	15,17	20,24	7,9	6,11	15,18	MATCH HN31
CCLC customer database	HN30	X,Y	10,12	11,12	11,12	13,16	28	16	12,13	10	15,17	20,24	7,9	6,11	15,18	MATCH HN30

Source	Sample_Name	AMEL	CSF1PO	D13S317	D16S539	D18S51	D21S11	D3S1358	D5S818	D7S820	D8S1179	FGA	TH01	TPOX	vWA	Comments
Defeng Deng	UM22A	X	10	8,12	9,11	18	28	16	8,12	8,9	11,13	22,24	6	8,11	18	
Clinical Cancer Research	UM-SCC-22/22A	X	10	8,12	9,11				12	8,9			6	8,11	15,18	
Head Neck 2010 April 32(4): 417-426	UM-SCC-22/22A	X		8,12		18	28	16	12	8,9	11,13	22,24			15,18	MATCH

Source	Sample_Name	AMEL	CSF1PO	D13S317	D16S539	D18S51	D21S11	D3S1358	D5S818	D7S820	D8S1179	FGA	TH01	TPOX	vWA	Comments
Defeng Deng	UMSCC11B	X	7	14	12,14	16	28	16	11	11	15	19	7	8	16,18	
Clinical Cancer Research	UM-SCC-11	X	7	14	12,14				11	11			7	8	16,17,18	
Head Neck 2010 April 32(4): 417-426	UM-SCC-11	X		14		16	28	16	11	11	12,15	19,24			16,17,18	MATCH

CCAC database is a comprehensive databases of short tandem repeat (STR) profiles, The database includes profiles from 20 public database/ publications and the cell lines developed by MD Anderson research labs.
The database contains over 5000 unique human cancer cell line STR profiles, one of the largest cancer cell line STR online search database in the world

Annual cell line authentication is required by UTMDACC INSTITUTIONAL POLICY # ACA1044
