# Supplementary figures and images for: Targeted Variant Assessments of Human Endogenous Retroviral Regions in Whole Genome Sequencing Data Reveal Retroviral Variants Associated with Papillary Thyroid Cancer
Source: Microorganisms. 2024 Nov 27;12(12):2435. doi: 10.3390/microorganisms12122435 (PMC11679660; doi:10.3390/microorganisms12122435)

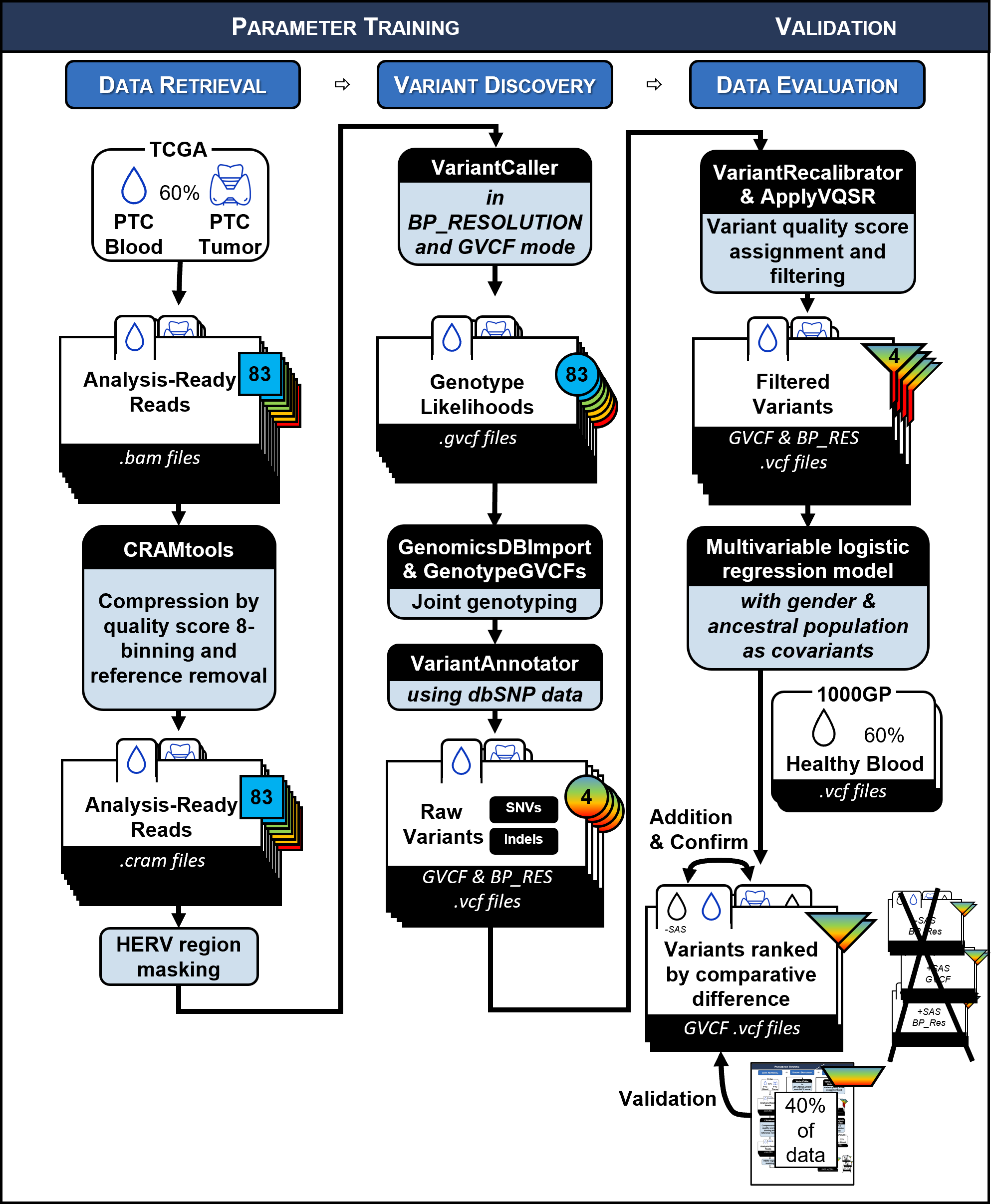

Supplement: Supplementary file 1 [file microorganisms-12-02435-s001.zip › Supplemental Figure S1. Variant call pipeline.png]

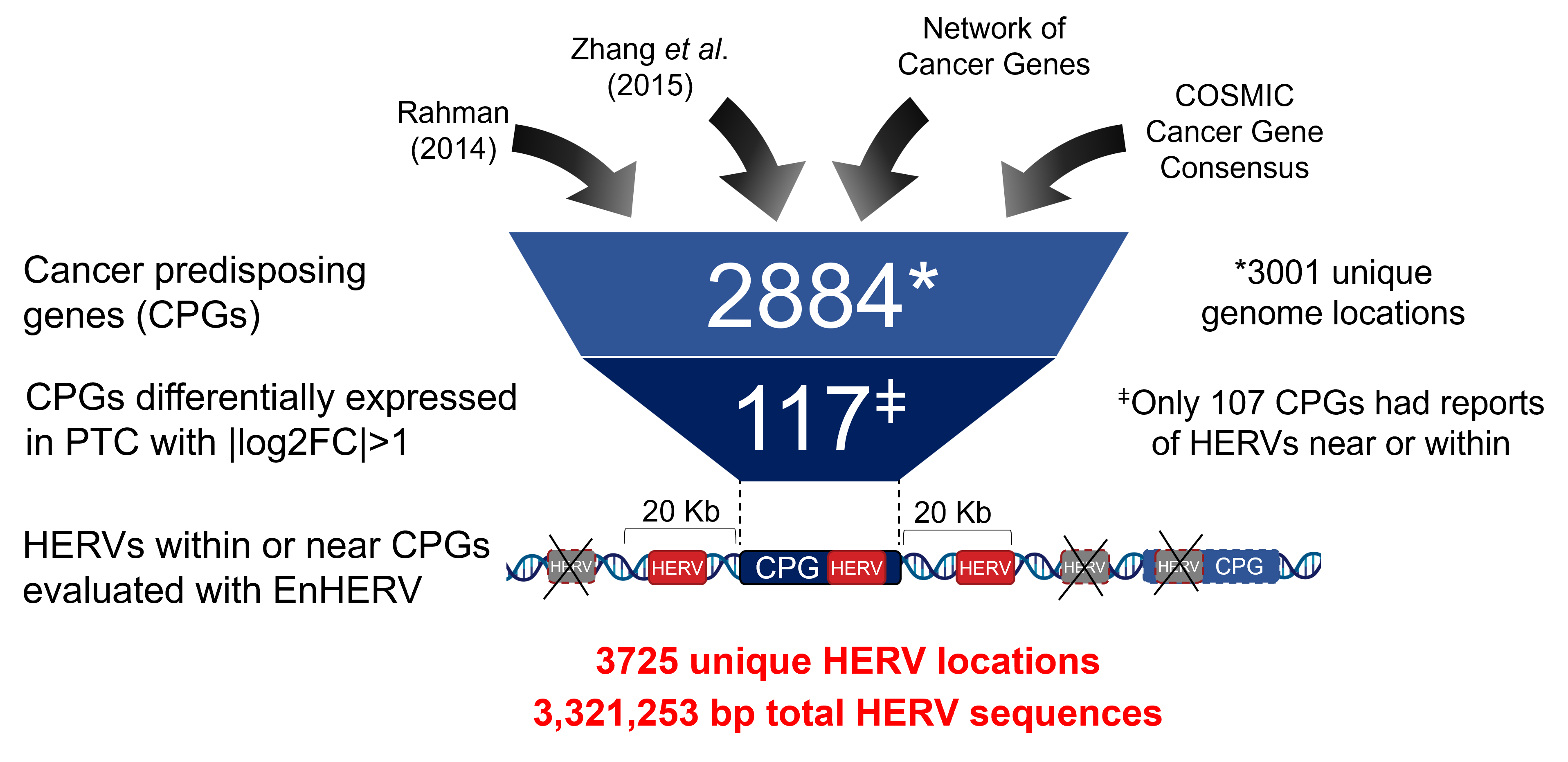

Supplement: Supplementary file 1 [file microorganisms-12-02435-s001.zip › Supplemental Figure S2. Figure Extraction of HERVs within 20kb radius of CPGs.png]

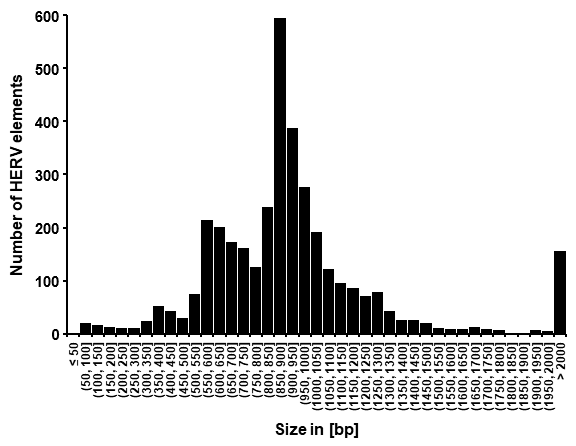

Supplement: Supplementary file 1 [file microorganisms-12-02435-s001.zip › Supplemental Figure S3. Size distribution of HERVs near of within CPGs.png]

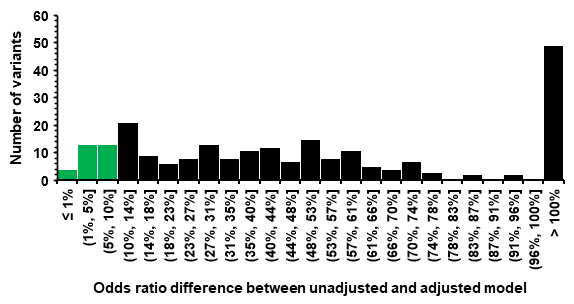

Supplement: Supplementary file 1 [file microorganisms-12-02435-s001.zip › Supplemental Figure S4.a Distribution of odds ratio changes for variants with significant p-values from the training set BLOOD.png]

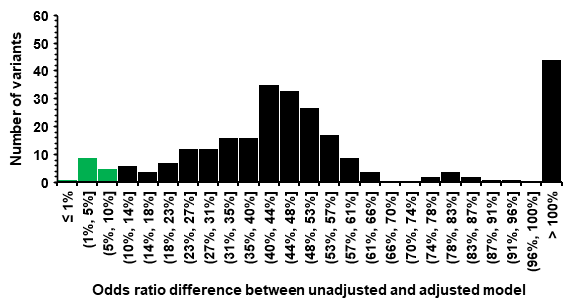

Supplement: Supplementary file 1 [file microorganisms-12-02435-s001.zip › Supplemental Figure S4.b Distribution of odds ratio changes for variants with significant p-values from the training set TUMOR.png]

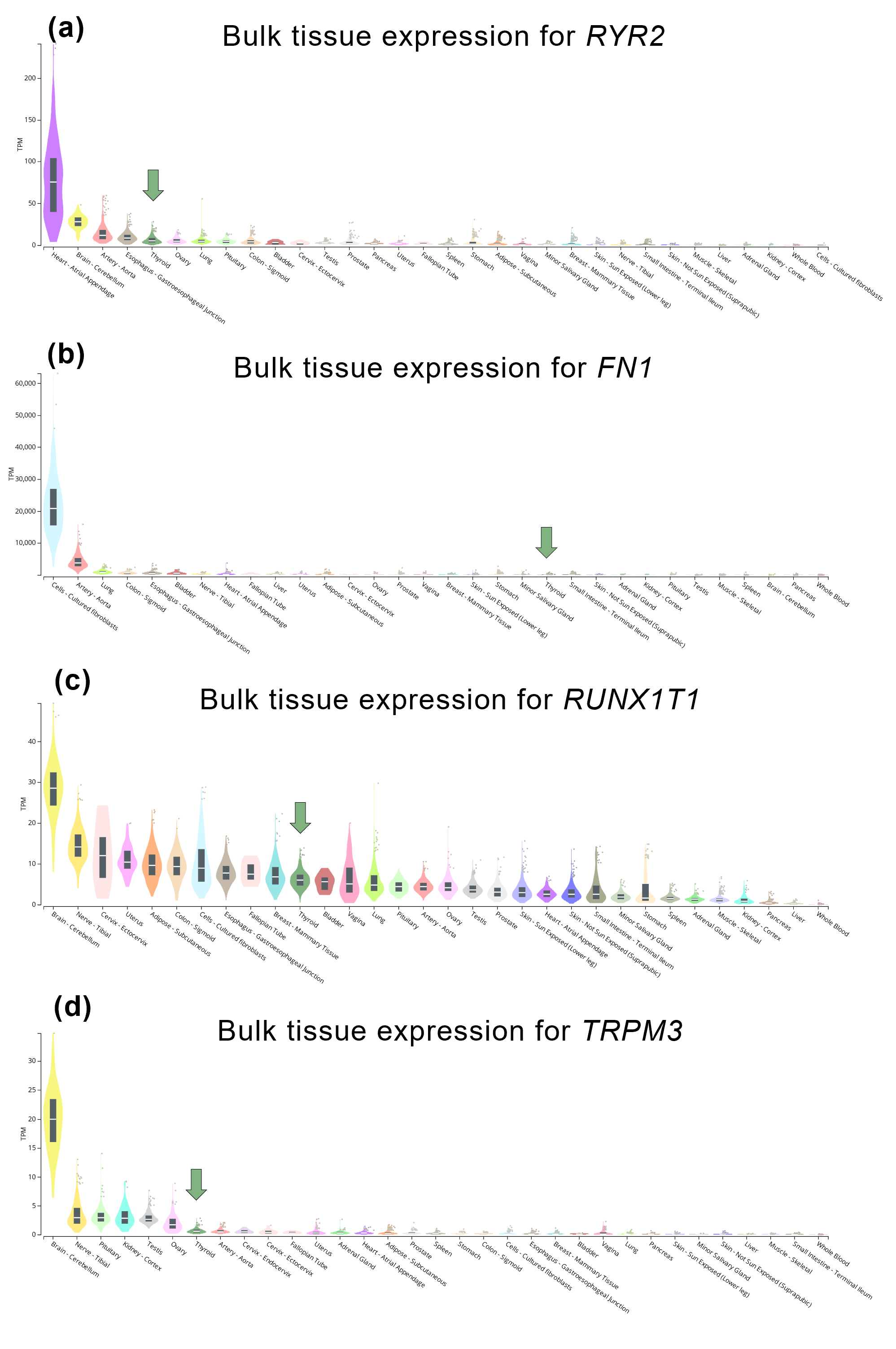

Supplement: Supplementary file 1 [file microorganisms-12-02435-s001.zip › Supplemental Figure S5.a-d GTEx results common variants.png]

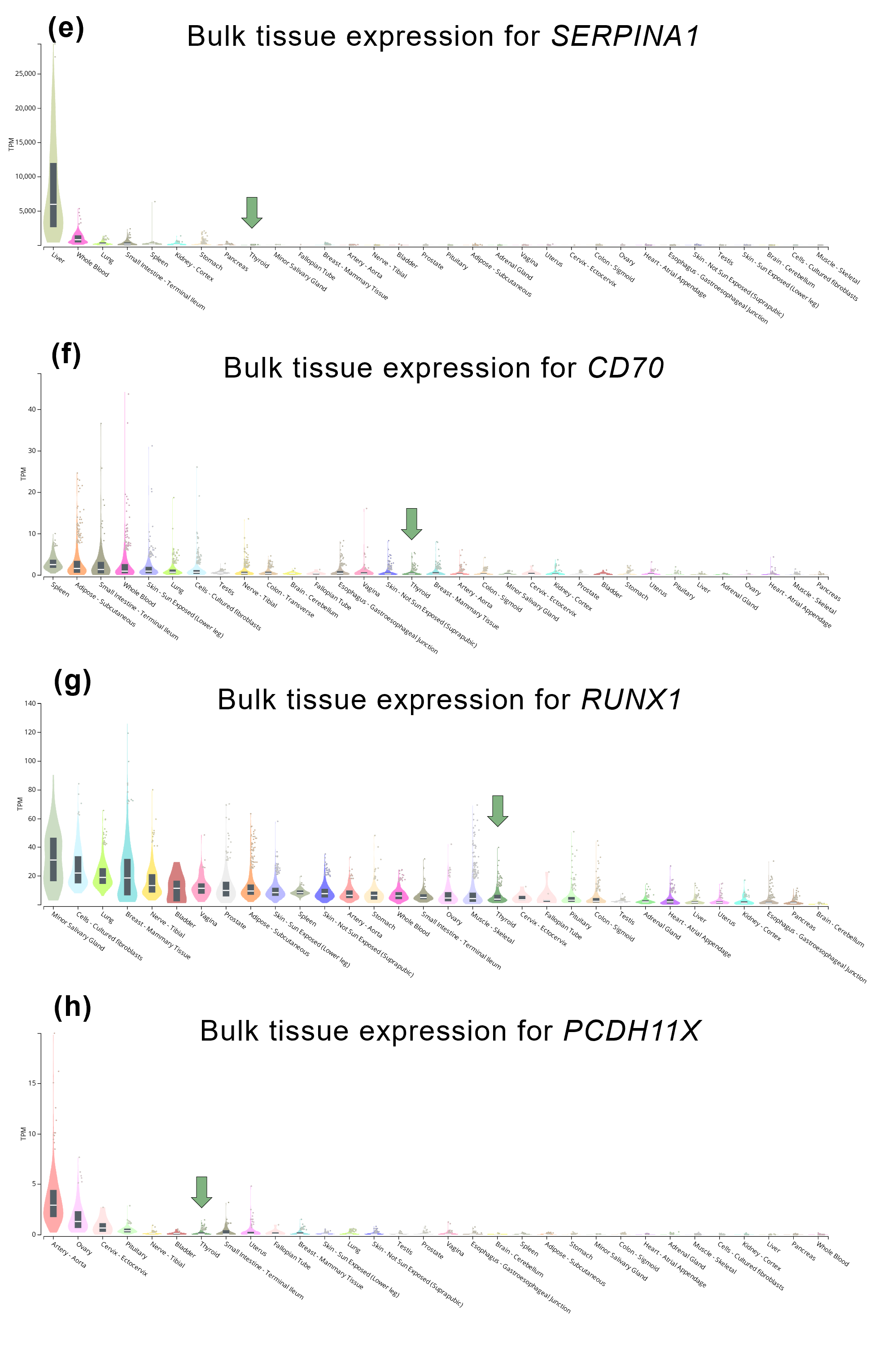

Supplement: Supplementary file 1 [file microorganisms-12-02435-s001.zip › Supplemental Figure S5.e-h GTEx results common variants.png]

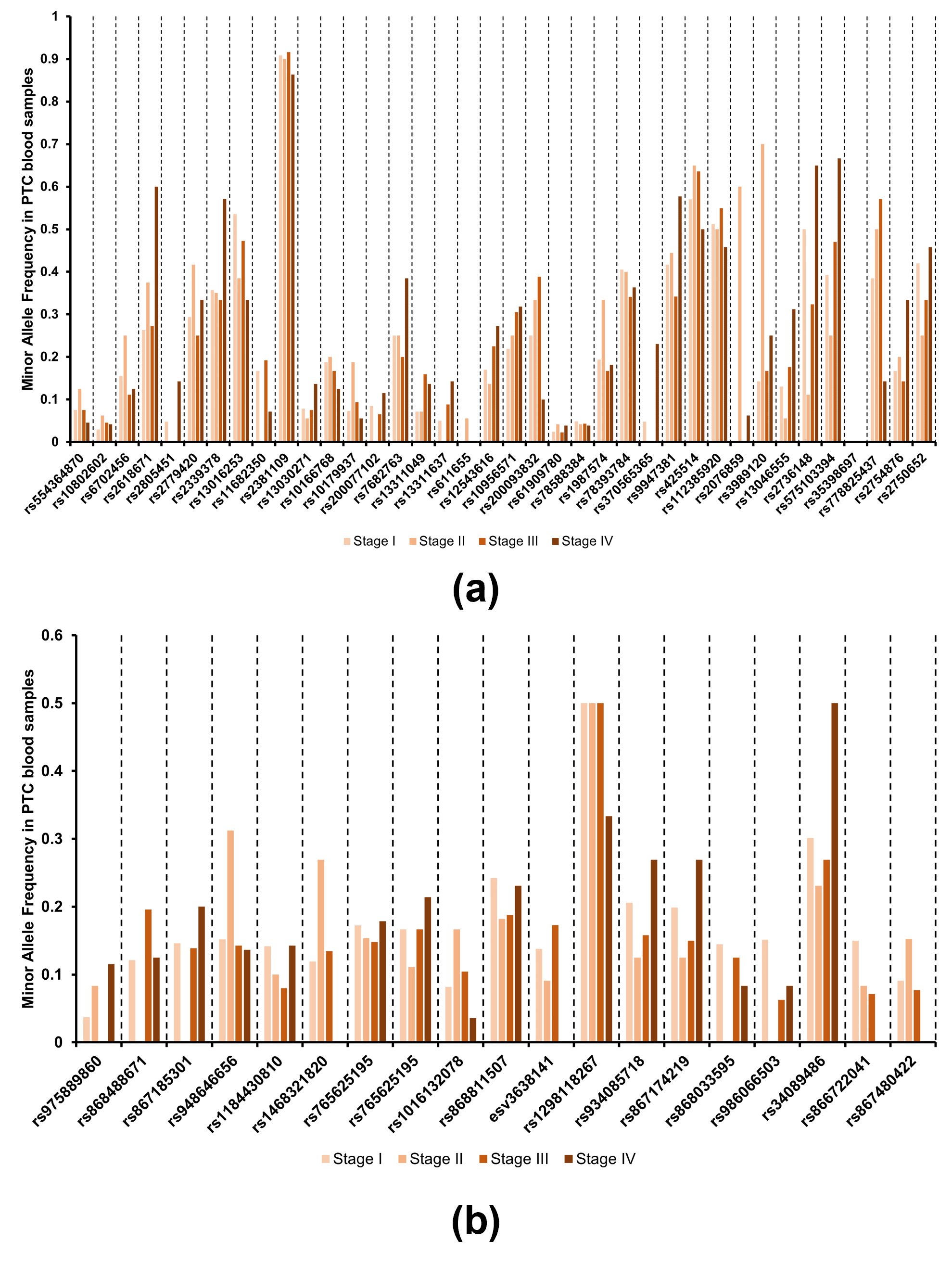

Supplement: Supplementary file 1 [file microorganisms-12-02435-s001.zip › Supplemental Figure S6. Minor allele frequencies by Stage.png]

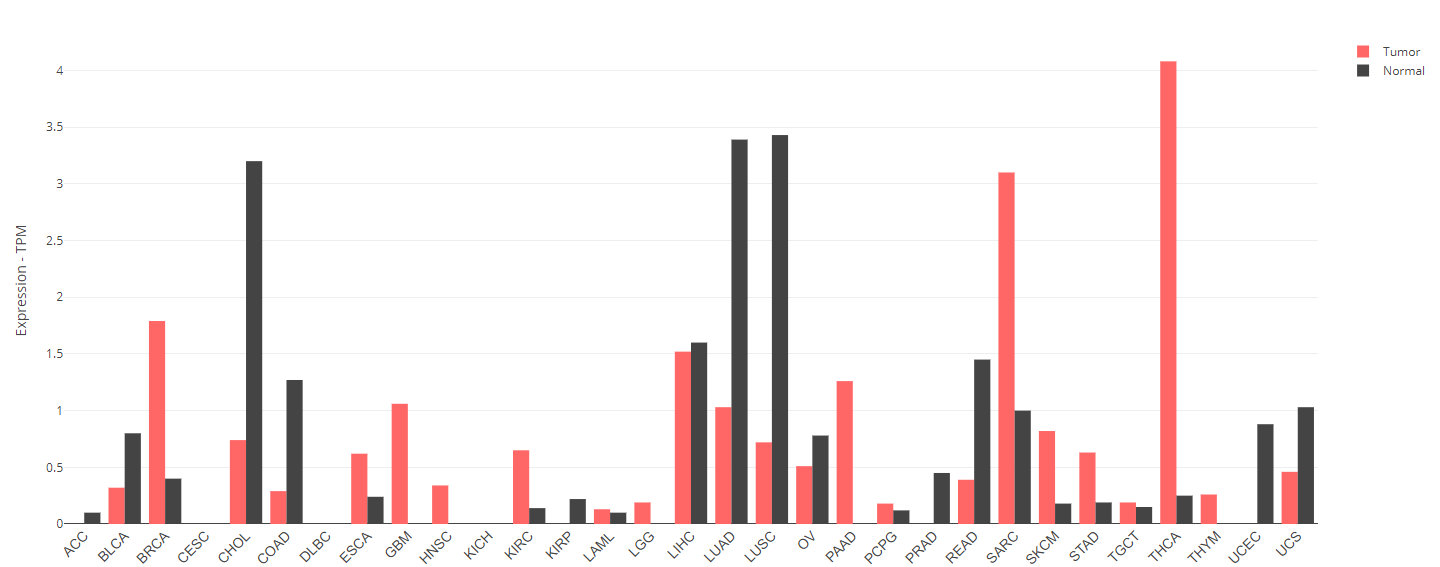

Supplement: Supplementary file 1 [file microorganisms-12-02435-s001.zip › Supplemental Figure S7.a Isoform expression profile of ENST00000460217.1.png]

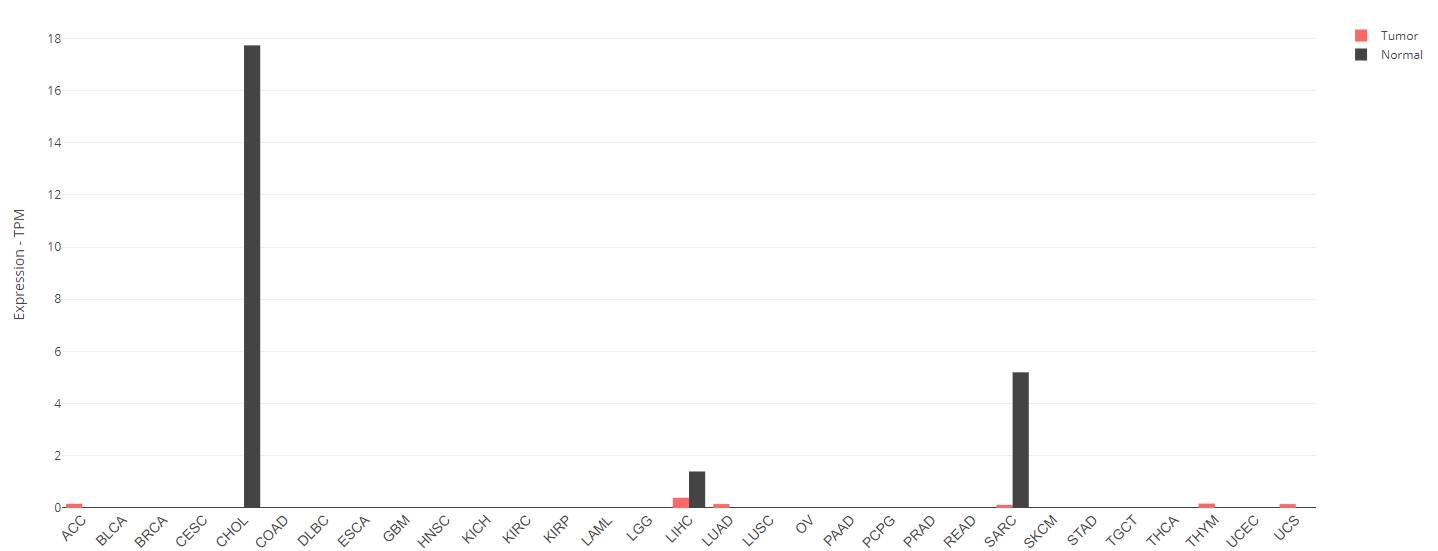

Supplement: Supplementary file 1 [file microorganisms-12-02435-s001.zip › Supplemental Figure S7.b Isoform expression profile of ENST00000438981.1.png]

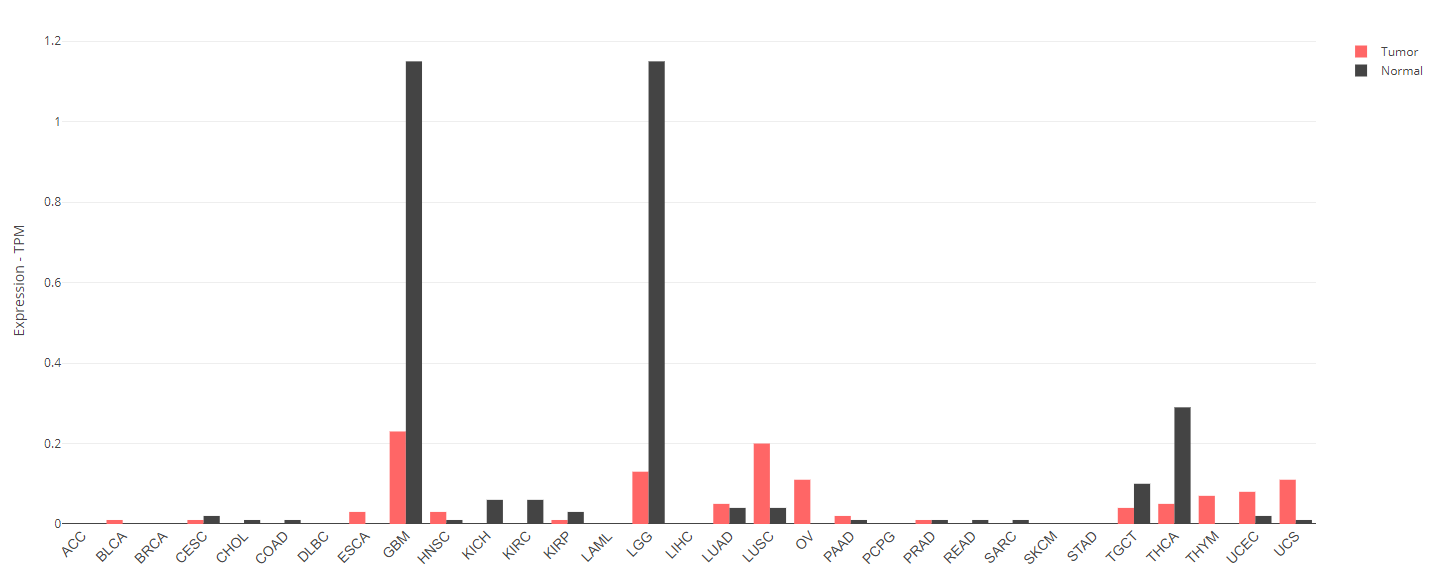

Supplement: Supplementary file 1 [file microorganisms-12-02435-s001.zip › Supplemental Figure S8.a Isoform expression profile of ENST00000619298.png]

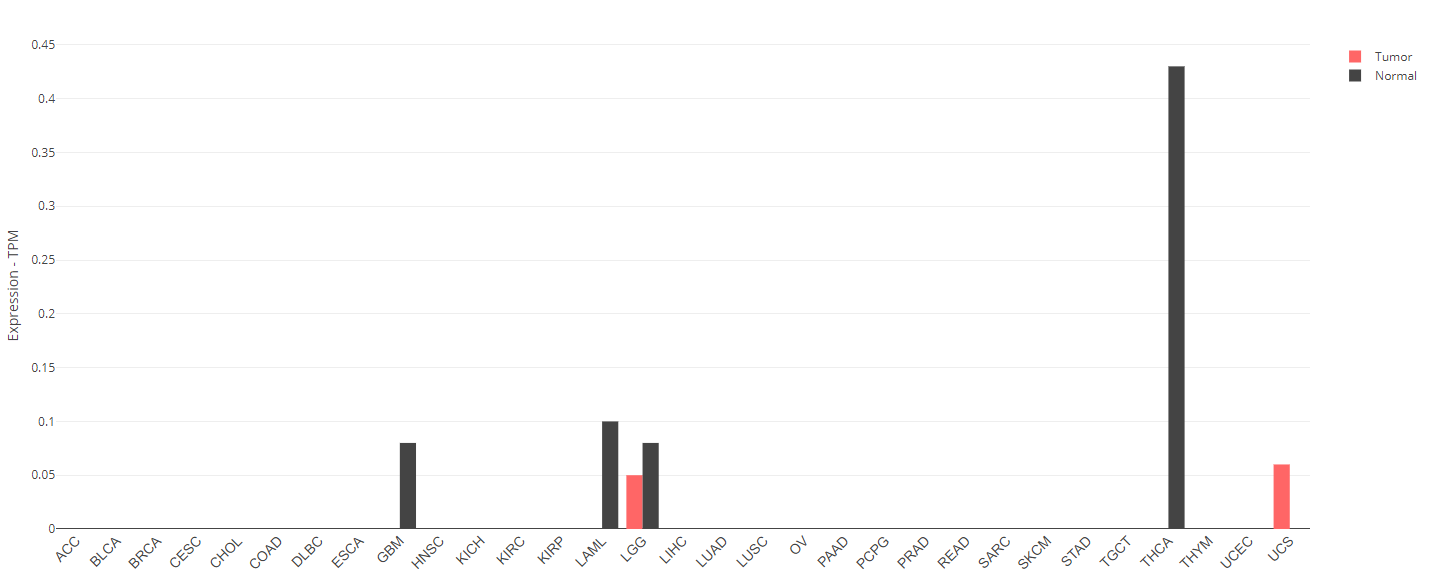

Supplement: Supplementary file 1 [file microorganisms-12-02435-s001.zip › Supplemental Figure S8.b Isoform expression profile of ENST00000525047.1.png]

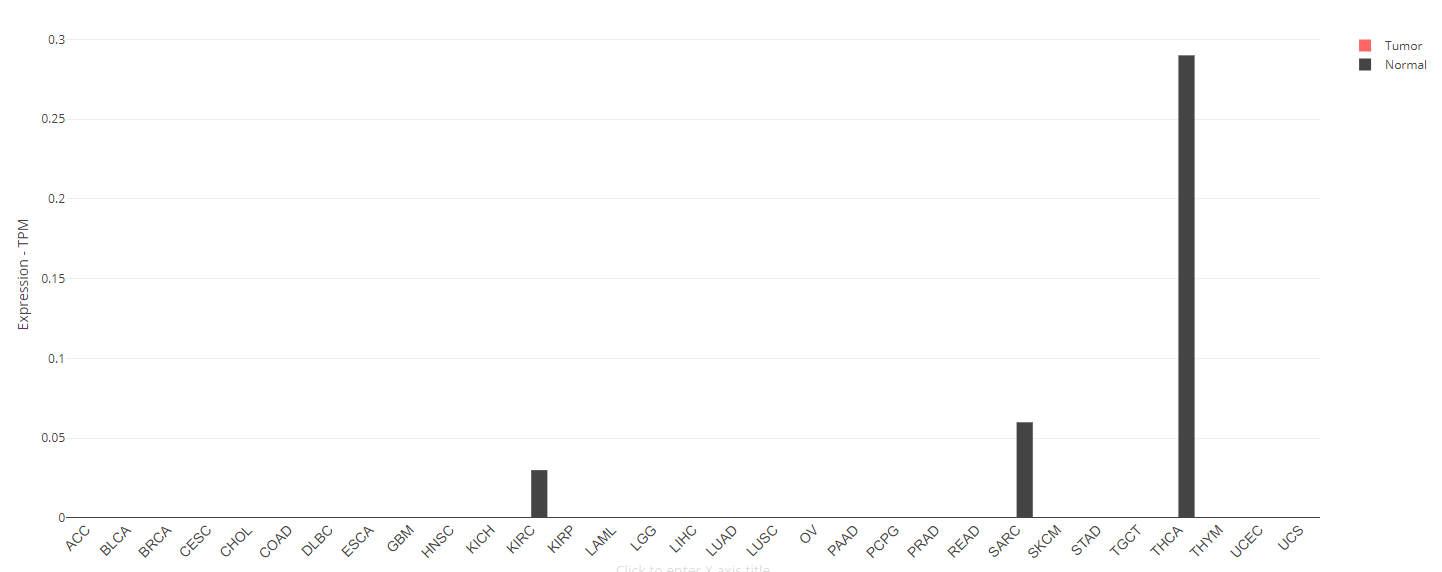

Supplement: Supplementary file 1 [file microorganisms-12-02435-s001.zip › Supplemental Figure S8.c Isoform expression profile of ENST00000524871.5.png]

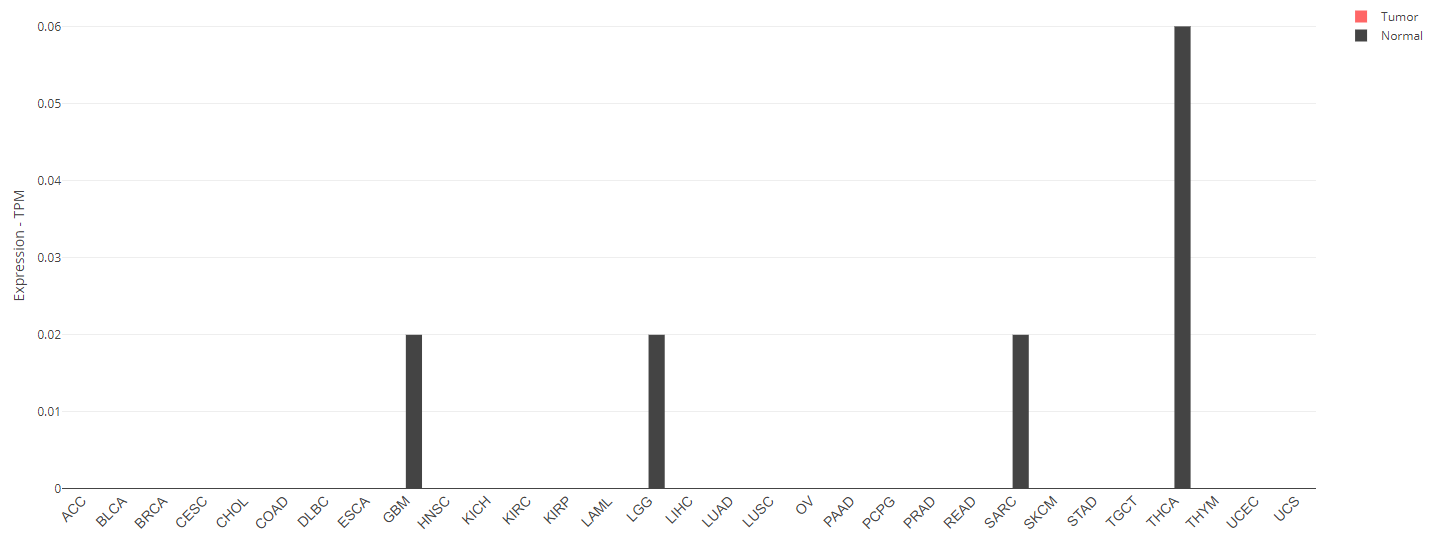

Supplement: Supplementary file 1 [file microorganisms-12-02435-s001.zip › Supplemental Figure S8.d Isoform expression profile of ENST00000528727.5.png]
